# Structure and Energetics of Chemically Functionalized Silicene: Combined Density Functional Theory and Machine Learning Approach

**DOI:** 10.3390/ma18225228

**Published:** 2025-11-19

**Authors:** Paweł Wojciechowski, Andrzej Bobyk, Mariusz Krawiec

**Affiliations:** 1 Institute of Physics, Maria Curie-Skłodowska University in Lublin, Pl. M. Curie-Skłodowskiej 1, 20-031 Lublin, Poland; mariusz.krawiec@umcs.pl; 2 Institute of Computer Science and Mathematics, Maria Curie-Skłodowska University in Lublin, ul. Akademicka 9, 20-031 Lublin, Poland; andrzej.bobyk@mail.umcs.pl

**Keywords:** silicene, adsorption, density functional theory, machine learning, surface functionalization

## Abstract

It is crucial to control and comprehend the interaction between elemental adsorbates and two-dimensional materials to drive future generations of electronic, sensing, and energy applications. One such material, particularly interesting from the perspective of tunability, is silicene—the silicon-based cousin of graphene. In this work, we investigate nearly 2000 atomic adsorption models on silicene via a combination of density functional theory (DFT) and machine learning (ML). Different systems with varied adsorption geometries, element identities, and surface coverages were optimized using spin-polarized DFT, and the most stable configurations were selected based on adsorption energy. This information was used to train various ML models, including tree-based models and artificial neural networks, to predict adsorption geometry (classification) and adsorption energy (regression). The current hybrid DFT + ML approach provides a transferable framework for high-throughput screening of element-functionalized silicene and other 2D surfaces, which is of immense importance in directing surface modification strategies in electronic and catalytic device engineering.

## 1. Introduction

One of the most promising functional surface engineering materials is silicene, a two-dimensional (2D) silicon allotrope with a buckled honeycomb structure. As a member of the Xene family, silicene possesses high carrier mobility and band tunability, rendering it ideal for nanoelectronics, chemical sensing, and energy storage [1,2,3,4,5,6,7,8]. One of the most intriguing properties of silicene, which it shares with its carbon-based predecessor and which makes these materials valuable in materials science, is the ease of functionalization. There are multiple possible mechanisms of silicene functional tuning with examples such as the application of electric field (which opens a band gap [9] and induces a topological phase transition [10]; such effects were absent in graphene and emerge only in bilayer structures [11]), charge doping (which changes the lattice parameters and buckling depending on the polarity [12]), or application of strain (which shifts the band structure perpendicular to the k-axis, leading to a self-doping effect [13]). Among the functionalization mechanisms, one that attracts the most attention is through the chemical modification—given the sp^2^-sp^3^ bond hybridization and general silicon affinity for pure sp^3^ bond, the chemical reactivity of silicene is much higher than in the case of graphene. An additional profit of such a process is the increased stability of the system, again thanks to the sp^3^ hybridization. In the set of possible approaches to chemical modification (i.e., defect engineering, atomic substitution, or substrate choice), one that is of special interest is atomic adsorption, thanks to its relative simplicity and still wide possible range of structure modification.

Several computational pathways can be used to investigate the physicochemical properties of adsorbate/substrate systems. Among them, density functional theory (DFT) is a widely used method that offers an accessible route to probe the electronic structure of complex many-body systems and reconciles accuracy with computational efficiency. Since its first construction by Hohenberg, Kohn, and Sham [14,15], various exchange–correlation functionals have been developed to capture complex electron interactions and enable predictive modelling across a wide range of systems, from nanomaterials study [16] and reaction mechanisms [17] to surface engineering [18], photocatalysis [19], and energy conversion technologies [20].

Simultaneously, machine learning (ML) has recently emerged as a revolutionizing tool in materials science [21,22,23,24]. ML models, trained on large datasets from experiments or simulations, are capable of rapid prediction of material properties and structure–property relationship identification with a big reduction in the design cycle. ML models in surface science have been utilized to predict adsorption energies, identify active sites, and support large-scale screening for catalytic [25], mechanical [26], and sensing applications [27].

Both of the above-mentioned techniques are characterized by their own set of advantages and disadvantages. While DFT enables quantitative insight into adsorption mechanisms and energetics, its computational cost inhibits high-throughput discovery, especially in the context of large configurational spaces [28]. In contrast, ML is generalizable and rapid but requires high-quality training data, and studying small datasets demands specialized approaches [29]. Surprisingly, the drawbacks of these two methods are not cumulative—instead, they appear complementary and represent a phenomenal paradigm when paired together: DFT delivers reliable training data, and ML enables rapid prediction of properties for untested systems [30]. Such an approach was successfully utilized in a variety of topics, with examples such as thermodynamics of alloys [31], heterogenous catalysis [32], and photovoltaics [33].

In this work, we present the first comprehensive combined DFT + ML study focused on atomic adsorption on silicene. While ML-assisted adsorption studies have been reported for other two-dimensional materials [34,35,36], silicene—despite its technological relevance—has not been systematically explored in this manner. We performed an extensive DFT investigation covering 96 elemental adsorbates (from hydrogen to curium) across five adsorption coverages and several surface geometries, identifying the most stable configurations for each element. The resulting dataset, encompassing nearly the entire periodic table, was complemented with tabulated atomic descriptors such as ionization energy and electronegativity and subsequently used to train multiple ML algorithms.

The main aspect of this study was to determine which of the selected descriptors are most crucial in the training process, i.e., which parameters have the highest significance in the selection/prediction process. For that, only properties that are easily accessible in an encyclopedia or physical tables have been chosen. Based on this, it is possible to ascertain whether one could accurately simulate (e.g., using our model) complex phenomena such as elemental adsorption using only the most fundamental parameters.

The resulting models accurately predict both adsorption energies and preferred binding sites (exceeding 95% accuracy), demonstrating that indeed a minimal set of easily accessible descriptors can reproduce complex DFT adsorption simulations. This study therefore provides both a large-scale reference dataset and a generalizable framework for predicting the physicochemical properties of nanostructures from limited input data, as well as pointing out which descriptors are meaningful in the machine training process.

## 2. Methods

**Computational Details.** Density functional theory (DFT) first-principles calculations were performed within the spin-polarized Perdew–Burke–Ernzerhof (PBE) generalized gradient approximation (GGA) [37] for the exchange–correlation functional in the Vienna Ab initio Simulation Package (VASP) [38,39]. Core electrons were handled using the projector-augmented wave (PAW) approach [40]. Plane-wave energy cutoff of 350 eV was consistently applied across all calculations. Electronic self-consistency was achieved with a total energy convergence threshold of 10^−7^ eV. We have built silicene supercells of sizes m × m (with m = 1, 2, 3, 4, 5) containing one adatom to explore adsorption behaviour under different surface concentrations of doping, which were measured in terms of N = (2 m) − 1 × 100%. The Brillouin zone of the 1 × 1 unit cell was sampled using a 16 × 16 × 1 Monkhorst–Pack k-point mesh that encompassed the Γ point [41], which was adjusted accordingly for larger supercells to ensure computational precision.

The silicene has been modelled by a single layer of Si atoms arranged in the honeycomb pattern. A vacuum region has been added to separate periodic images of the layer, thus avoiding the interaction between them. Therefore, the unit cell in the z direction was 20 Å long. The low-buckled atomic configuration, characteristic of the isolated silicene layer, was assumed as the initial guess during the geometry optimization. In the search for the lowest energy structural model of silicene decorated by adatoms of various chemical elements, different adsorption sites have been carefully checked. In that process, adatoms were initially placed ~3 Å above the silicene layer, and then allowed to move in all directions. Of course, the silicene layer was also subjected to the geometry relaxation. The atomic positions were optimized by a conjugate gradient method until the largest force in any direction was below 0.01 eV/Å.

**Machine learning.** In addition to our DFT studies and to enable effective prediction of adsorption properties, we have developed and trained machine learning (ML) models for adsorption site identification and prediction of adsorption energy. These models were run using the Scikit-learn Python library (version 1.3.2) [42] (Gradient Boosted Trees [43] algorithm) complemented by stand-alone codes using XGBoost (version 1.7.6) [44] and LightGBM (version 4.5.0) [45]. In addition, two artificial neural networks, one for classification and the other for regression purposes, were developed employing TensorFlow (version 2.18.0) [46] and Keras (version 3.8.0) [47] frameworks. The dataset included adsorption scenarios with all the entities present on four different silicene substrates to ensure the models’ broad applicability in cases of diverse adsorbates. Data pre-processing entailed standard scaling (z-score normalization) and one-hot encoding (for coverage variables).

The entire dataset was primarily separated using a 75:25 split. The smaller part (25%) of the dataset was reserved exclusively as the test set for final performance evaluation (i.e., models had no access to this part during training step) in order to prevent potential data leakage. The larger portion (75%) was designated for combined training and validation, with 5-fold cross-validation being used for hyperparameter tuning. Hyperparameters were optimized with a random search strategy over 24 parameter settings, as stated in Supplementary Materials (Table S4).

**Visualization.** Atomic structure models presented in this work were visualized using VESTA 3 software [48].

## 3. Results and Discussion

### 3.1. Input Data Preparation

**Figure 1:**
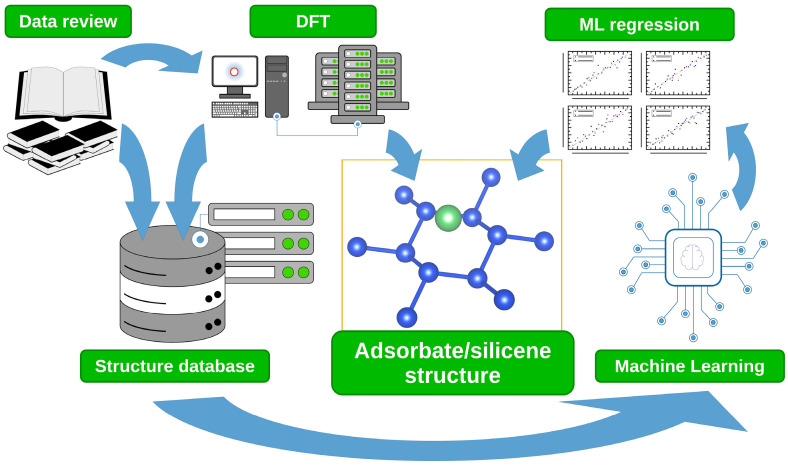
Workflow diagram summarizing steps performed in this study.

Figure 1 presents a simplified workflow diagram summarizing the investigation described in this paper. Two initial steps—encyclopedic data collection and DFT calculations—were performed concurrently. For data collection, several elemental properties were selected as input parameters for subsequent analysis: atomic covalent radii [49], ionization energy [50], electronegativity [51], and valency [52] (in this study “number of valence electrons”, not “number of chemical bonds”). Among electronegativity scales, the Pauling electronegativity was primarily used based on its widespread acceptance; however, it is not defined for every element, as some lack the stable covalent compounds required for Pauling’s methodology. In such cases, alternative electronegativity scales were employed, namely the Allred–Rochow scale [53] (based on the electrostatic interaction between the nucleus and the valence electrons) and the Mulliken scale [54,55] (based on electron affinity and ionization potential). The entire dataset of encyclopedic input data is provided in Supplementary Materials (Table S1).

**Figure 2:**
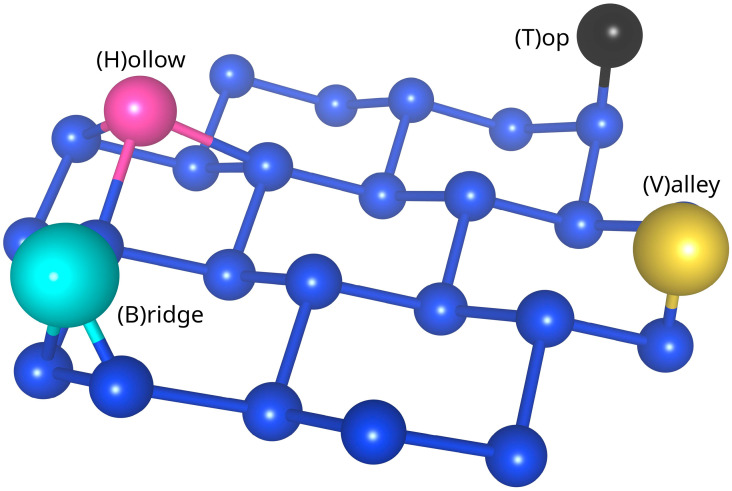
Schematic depiction of the adsorption geometries analyzed in this work: T (top), V (valley), B (bridge), and H (hollow).

Multiple adsorbate/silicene models were constructed, each characterized by three parameters: adsorbed element, adsorption geometry, and surface coverage. The adsorption geometries under consideration are presented in Figure 2 and include the following: adsorption atop the lower silicon atoms (“V” for valley), atop the higher silicon atoms (“T” for top), above silicon–silicon bonds (“B” for bridge or bond), and at the centre of the silicene hexagon (“H” for hollow or hexagon). The studied surface coverages range from 1 × 1 (one atom per silicene unit cell) to 5 × 5 (one atom per 25 silicene unit cells). Assuming that full coverage results in occupation of all possible adsorption sites, these coverages correspond to 100%, 25%, 11.1%, 6.25%, and 4%, respectively. This work does not consider nonsymmetrical reconstructions (e.g., 2 × 1) nor multi-site adsorption (e.g., simultaneous occupation of T and V sites).

### 3.2. DFT Calculations

**Figure 3:**
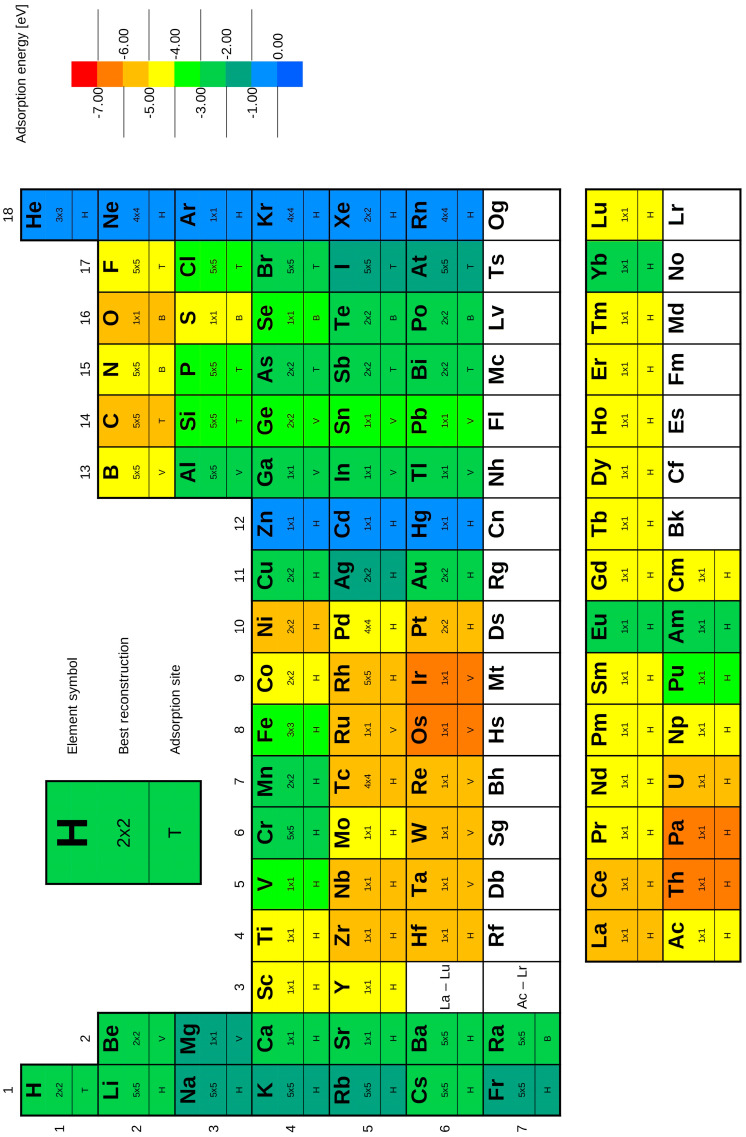
Summary of DFT results in the form of the periodic table, including best reconstruction (i.e., coverage) and adsorption site. Colour scale represents the adsorption energy.

The adsorbate/silicene models described in the previous section were fully optimized using DFT. For each element, the structure with the lowest total energy was selected as the most stable adsorption configuration. The adsorption energy *E_a_* was calculated according to the following formula:

$$E_{a}=E_{0}\left(system\right)-\left(E_{0}\left(silicene\right)+E_{0}\left(adatom\right)\right)$$

where *E*_0_(*system*) is the total energy of the optimized structure, *E*_0_(*silicene*) is the total energy of pristine silicene, and *E*_0_(*adatom*) is the total energy of an isolated adsorbate atom. The graphical summary of the adsorption energies, most favourable adsorption site, and optimal reconstructions is presented in Figure 3, where adsorption energy is indicated by a colour scale ranging from red (highest *E_a_*) to blue (lowest *E_a_*).

An important consideration is the inherent precision of DFT calculations. For several elements—particularly noble gases, but also several transition metals such as Cr and Fe—the adsorption geometries were almost degenerate, with energy differences between the most stable and second most stable structures <10 meV. Such small differences approach or fall below the expected precision limits of DFT, which depend on the choice of pseudopotentials and computational parameters [56,57]. Detailed DFT results, including input parameters and separate breakdown by coverage, are provided in the Supplementary Materials (Tables S1 and S2).

Direct comparisons with previous multi-element adsorption studies on silicene remain challenging due to the limited availability of systematic investigations across varying supercell sizes. Nguyen et al. [58] reported halogen adsorption on silicene, finding that all group 17 elements preferentially adsorb at the top (T) site regardless of coverage, consistent with our results. In their study, the 4 × 4 supercell was the largest considered and most favourable, whereas we observe a further stabilization at the 5 × 5 coverage, reinforcing the trend that for halogen elements adsorption energy generally increases (becomes more favourable) with decreasing coverage. Interestingly, chlorine remains one of the several elements which adsorption on silicene was investigated experimentally. Li et al. [59] reported chlorine adsorption on epitaxial silicene grown on Ag(111) using scanning tunnelling microscopy (STM). Their observations confirm Cl adsorption at T sites across varying doses and silicene reconstructions, even when near-saturation coverage affects the buckling of silicene, which is in line with our computational predictions.

Other studies tend to focus on fixed silicene supercell sizes. For example, Sun et al. [60] examined third period elements up to copper (along with H, Li, Be, Na, and Mg) adsorbed on a 6 × 6 silicene supercell. The adsorption geometries reported largely agree with those found here for the 5 × 5 coverage, except for discrepancies in Ca, Ti, and Fe. Lin and Ni [61] studied 15 different elements, mainly transition and alkali metals, on a 4 × 4 supercell and found adsorption sites consistent with our 4 × 4 results. Li et al. [62] similarly investigated rare metal adsorption on 4 × 4 silicene, confirming comparable adsorption behaviours. Kaloni and Schwingeschlögl [63] explored the adsorption of Au, Hg, Tl, and Pb on a 4 × 4 silicene supercell. While Au, Hg, and Tl adsorption sites agree well with our results, Pb shows a notable discrepancy: Pb atoms preferentially adsorb at the valley (V) site in our study, whereas the referenced work reports hollow (H) site adsorption. The *E_a_* difference is approximately 340 meV in our calculations, compared to about 50 meV reported by Kaloni and Schwingenschlögl. This discrepancy may arise from differences in computational approximations and parameter choices.

### 3.3. ML Models Benchmarking

A range of ML algorithms were employed to construct predictive models for adsorbate/silicene system screening, including Gradient Boosted Trees (GBT), XGBoost (XGB), LightGBM (LGBM), Random Forests (RF), and a dedicated artificial neural network (ANN). All models were trained and validated using 5-fold cross-validation. Following model optimization, predictions were generated on the test subset of the dataset.

**Figure 4:**
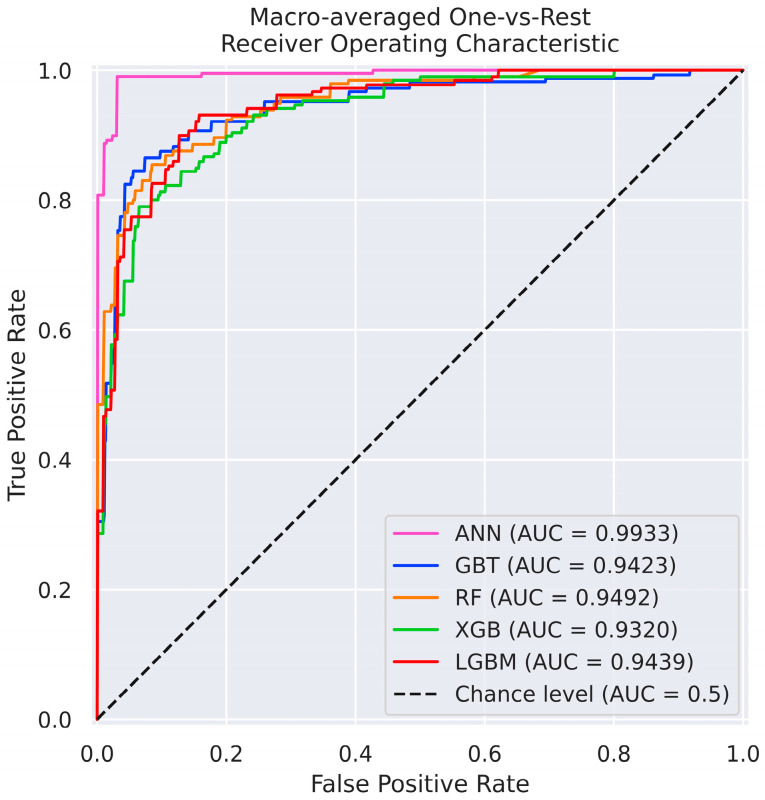
Receiver operating characteristic (ROC) plots for classification predictions on the test dataset with the corresponding area under curve (AUC) values, presenting prediction potential of ML-trained models.

The first predictive task involved classification of the most favourable adsorption geometry. Table 1 summarizes the macro-averaged performance metrics for all models, while Figure 4 presents a direct comparison of their receiver operating characteristic (ROC) curves and associated area under the curve (AUC) values. Definitions and equations of the evaluation metrics, as well as full confusion matrices, are provided in the Supplementary Materials. Among the tested models, the ANN demonstrated the highest overall accuracy, achieving over 96% across all performance measures and an ROC AUC approaching the ideal value of 1.0, indicating excellent discriminatory capability.

**Figure 5:**
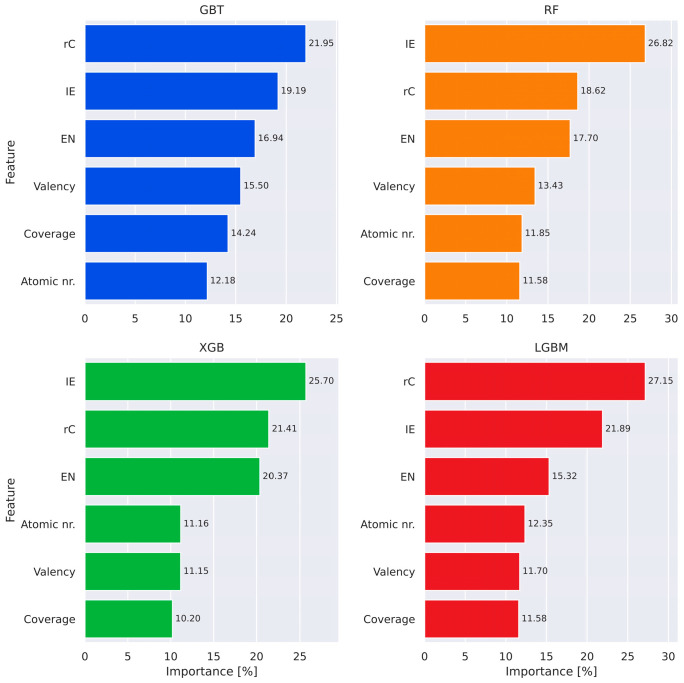
Feature importance (Shapley) plots for “classical” machine learning classification models, revealing the impact of chosen descriptors on the selection process.

Feature importance in the form of Shapley plots for the tree-based models is presented in Figure 5. As k-fold cross-validation was utilized, the Shapley plots were computed on the entire dataset. For all the algorithms, the highest contributing features were the covalent radius and the ionization energy, with electronegativity consistently ranking third. The beneficial effect of ionization energy is likely to be a result of its connection with chemical reactivity: both noble gases (plus Hg), which have the highest ionization energies, and alkali metals with low ionization energies preferentially adsorb in hollow (H) sites. Electronegativity distinguishes other extremes, such as halogens, which all adsorb at top (T) sites and possess the highest electronegativities, with alkali metals again occupying the low end of the scale.

The dominance of the covalent radius underscores the importance of geometric factors in adsorption site preference. Notably, nearly all elements with covalent radii above 150 pm—and approximately 75% of those exceeding 125 pm—adsorb preferentially at H sites, supporting a size-driven site selection mechanism. In contrast, atomic number and surface coverage exhibited minimal influence on classification performance, suggesting their limited role in determining adsorption geometry under the conditions considered.

Apart from classifying the best adsorption site, ML algorithms were individually trained to predict the adsorption energy (*E_a_*) of the adsorbate–silicene system. Table 2 reports an overview of the regression performance in the test set. Among the algorithms used, the LightGBM (LGBM) model gave the highest predictive accuracy with an R^2^ value greater than 0.98 and smallest mean absolute and root mean square errors.

**Figure 6:**
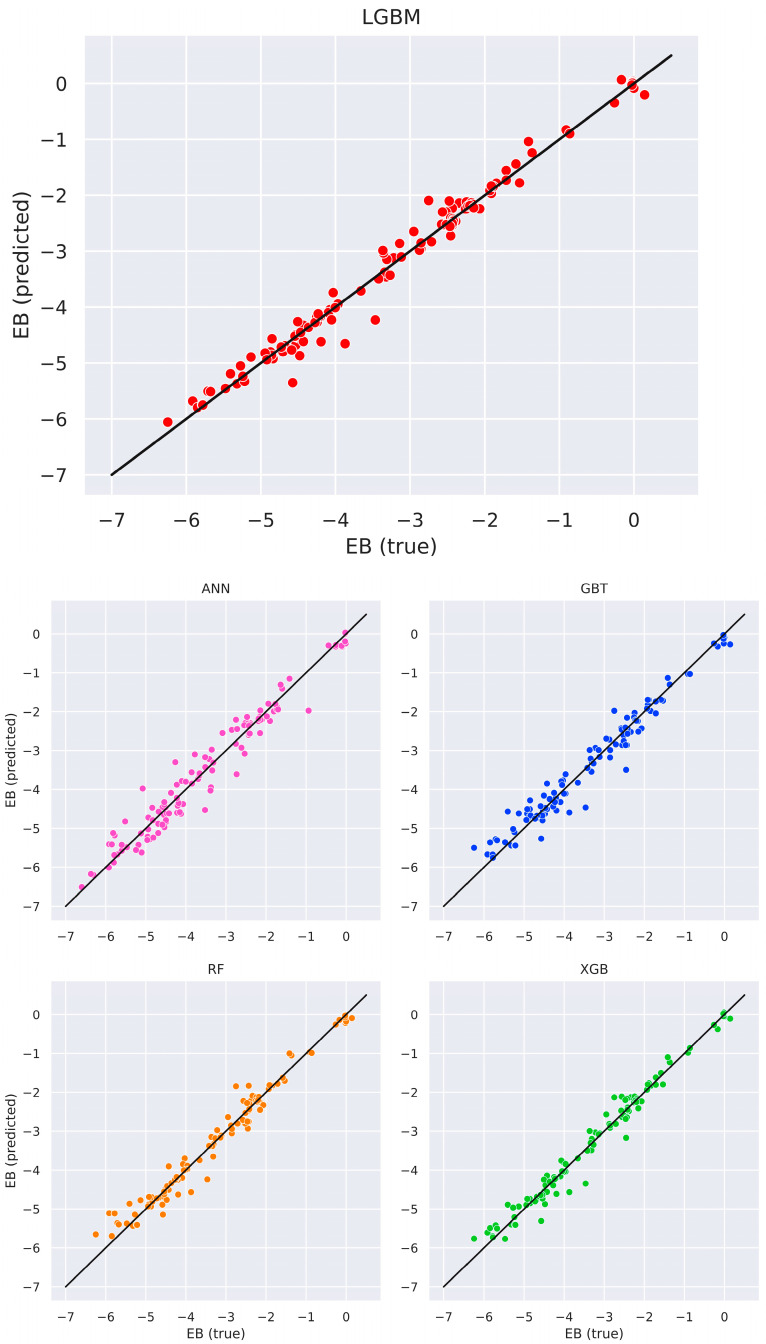
Scatter plots showing correspondence between actual values (in eV) of the adsorption energy (*E_a_*) and values predicted by the corresponding regression model on the test dataset.

It is to be noted, though, that all models achieved R^2^ > 0.95, validating high overall consistency and prediction reliability of adsorption energy regardless of the algorithm used. Validation phase performance metrics, presented in the Supplementary Materials (Table S6), reveal more even distribution across the models, albeit with slightly smaller R^2^ values. This suggests that the regression might be moderately sensitive to a specific data partition or chemical class, highlighting the case-dependent prediction nature of certain systems. Scatter plots of predicted versus actual *E_a_* values are displayed in Figure 6 and illustrate a high correlation between ML and DFT outputs.

**Figure 7:**
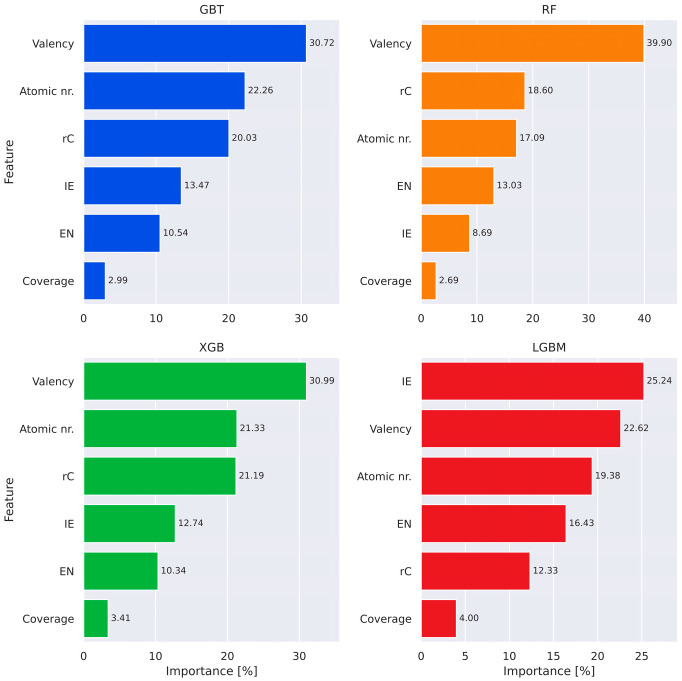
Feature importance (Shapley) plots for “classical” machine learning regression models, revealing the impact of selected descriptors on the prediction process.

Similarly to the classification task, feature contribution to prediction of adsorption energy was determined using Shapley analysis, as shown in Figure 7. In this case, the number of valence electrons was found to be the most important feature. This result aligns with chemical expectation since valence electrons are directly involved with bond formation and hence are expected to play an important role in adsorption energetics. Ionization energy, covalent radius, and atomic number were the other principal contributors (depending on the model used). The ionization energy, as stated in the discussion of feature importance of the adsorption site prediction, covers two extreme cases: noble gases (high ionization energy) and alkali metals (low ionization energy; less important in *E_B_* prediction). It is noteworthy that in the prediction process of the LGBM-based model the priorities were different, with ionization energy being the most significant descriptor and covalent radius the second last. The origin of such a discrepancy in feature importance between the models is unknown at present.

While the straightforward link between covalent radius and adsorption energy is complex, it has been used as a descriptor in ML-based investigations [64], with the general relation “smaller radius → stronger adsorption” (it must be underlined that this is only a general relation, not fundamental law, and therefore it is subjected to frequent exceptions among the elements). Atomic number directly relates to the periodic character of the elemental table, and such periodicity can be observed (Figure 3). On the other hand, surface coverage was the least effective parameter. This is consistent with the local nature of single-atom adsorption, which dominates the near atomic environment and less sensitively responds to global fluctuations in coverage.

## 4. Summary

An extensive exploration of the adsorption of elements on silicene was conducted under the assistance of first-principles density functional theory (DFT) calculations. These calculations were accompanied by a meticulous collection of atomic descriptors including covalent radius, ionization energy, electronegativity, and valence that collectively formed the basis of a structured database. This information was then used to train and validate a number of machine learning (ML) models, four based on various training algorithms and one on an artificial neural network (ANN), for two tasks: adsorption site classification and prediction of adsorption energy.

Among the classification models, ANN exhibited the best performance, with ROC AUC values >0.99 and high results across all metrics. On adsorption energy regression, the LGBM model was the most accurate with an R^2^ coefficient above 0.98. Noteworthy is the fact that all models had high predictive powers with ROC AUC > 0.93 and R^2^ > 0.95—this indicates that ML-trained models reliably predict adsorption energy and geometry regardless of the algorithm chosen, and are robust to specific data partition.

The key results of this study are related to the feature importance plots that reveal which of the analyzed descriptors have the most impact in classification/prediction processes, respectively. In the former, covalent radius and ionization energy have the highest weight in the decision process, with electronegativity being the third most significant parameter in every model. In the adsorption energy prediction model, the valency dominates, with covalent radius and atomic radius slightly less important—this is with the exception of the LGBM model, in which ionization energy gains significance at the cost of covalent radius. Notably, in almost every model, coverage is the least important parameter (with the exception of the GBT classification model, in which it is second least), underlining its limited influence on the single-atom adsorption process.

These results show that ML models, when trained on chemically relevant descriptors, can effectively contribute to the high-speed screening of adsorption systems. Such models offer a valuable addition to high-accuracy quantum mechanical methods, enabling the rapid identification of good candidates for targeted DFT investigation and rational material design. The insights gained in this research are valuable to a broad range of 2D surface functionalization applications. Reducing the cost of input for the prediction of adsorption behaviour may be particularly valuable for designing silicene-based sensors and electronic interfaces, and for catalytic systems design, where sensitive surface tuning is paramount. Furthermore, the approach outlined here is readily adaptable to other 2D systems such as germanene, phosphorene, or transition metal dichalcogenides with a material-optimizing protocol that can be generalized. The integration of data-driven models in high-throughput screening routines has the power to initiate fast discovery routes in surface science with predictive capability and physical insight.

## Figures and Tables

**Table 1:** Classification macro-average metrics on the test dataset for ML models, predicting the adsorption site.

	ACC	PPV	TPR	F1	ROC AUC
**ANN**	0.9667	0.9626	0.9782	0.9693	0.9933
**RF**	0.8333	0.8393	0.8322	0.8348	0.9542
**GBT**	0.8167	0.8152	0.8122	0.8092	0.9454
**LGBM**	0.7833	0.7549	0.7843	0.7670	0.9472
**XGB**	0.7583	0.7289	0.7843	0.7418	0.9374

ACC—accuracy. PPV—positive predictive value (precision). TPR—true positive rate (recall). F1—F1 score (harmonic mean of precision and recall). ROC AUC—area under the curve of the receiver operating characteristic.

**Table 2:** Regression metrics on the test datasets for ML models, predicting the adsorption energy.

	MAE	RMSE	R^2^
**LGBM**	0.1294	0.2012	0.9843
**XGB**	0.1567	0.2298	0.9796
**RF**	0.1745	0.2620	0.9734
**GBT**	0.2185	0.2984	0.9655
**ANN**	0.2438	0.3291	0.9586

MAE—mean absolute error. RMSE—root mean square error. R^2^—coefficient of determination.

## Data Availability

The original contributions presented in this study are included in the article/Supplementary Material. Further inquiries can be directed to the corresponding author.

## Supplementary Materials

The following supporting information can be downloaded at https://www.mdpi.com/article/10.3390/ma18225228/s1. 1. Summary of input data and DFT results; 2. DFT results for all analyzed coverages; 3. Graphical representation of DFT results for all coverages, in the form of periodic tables; 4. Confusion matrix, definitions of used performance metrics, and their equations; 5. Hyperparameters of the ML; 6. Confusion matrices of classification models; 7. Performance metrics on the validation dataset.
