# Supplementary material for: Structure and Energetics of Chemically Functionalized Silicene: Combined Density Functional Theory and Machine Learning Approach
Source: Materials (Basel). 2025 Nov 19;18(22):5228. doi: 10.3390/ma18225228 (PMC12654217; doi:10.3390/ma18225228)
Supplement: Supplementary file 1 [file materials-18-05228-s001.zip › materials-3945359-supplementary.pdf]

Structure and energetics of chemically functionalized silicene: combined density functional theory and machine-learning approach

Supplementary materials

Paweł Wojciechowski[1,], Andrzej Bobyk[2], Mariusz Krawiec[1]*

[1]Institute of Physics, Maria Curie-Skłodowska University in Lublin, Pl. M. Curie-Skłodowskiej 1, 20-031 Lublin, Poland

[2]Institute of Computer Science and Mathematics, Maria Curie-Skłodowska University, ul. Akademicka 9, 20-031 Lublin, Poland

*Corresponding author: Paweł Wojciechowski pawel.wojciechowski@mail.umcs.pl

Contents:

1. Summary of input data and DFT results

2. DFT results for all analysed coverages

3. Graphical representation of DFT results in the form of periodic tables

4. Error matrix, definitions of used statistical metrics and their equations

5. Hyperparameters of the ML

6. Confusion matrices of classification models

7. Statistical metrics on the validation dataset

1. Summary of input data and DFT results

Table S1 contains tally of encyclopedic data used for machine-learning, including: rC – covalent radii, IE – ionization energy, EN – electronegativity, E_a – adsorption energy. Several elements have more than one covalent radii – in those cases brackets describe which was used for further analysis (h.s. – high spin). The AR superscript in EN column means Allred-Rochow electronegativity is provided, M superscript – Mulliken electronegativity. Examples where different structures are almost degenerate energetically (i.e. difference between the best and second best systems are below 10 meV) are marked green.

Table S1. Summary of input data and DFT results.

Atomic nr.	Element	r_C [pm]	IE [eV]	EN	Valence	Coverage	Site	E_a
1	H	31	13.598	2.20	1	2x2	T	-2.157
2	He	28	24.587	5.50^{AR}	0	3x3	H	-0.009667
3	Li	128	5.392	0.98	1	5x5	H	-2.255
4	Be	96	9.323	1.57	2	2x2	V	-2.453
5	B	84	8.298	2.04	3	5x5	V	-4.847
6	C	76 (sp3)	11.260	2.55	4	5x5	T	-5.797
7	N	71	14.534	3.04	5	5x5	B	-4.680
8	O	66	13.618	3.44	2	1x1	B	-5.914
9	F	57	17.423	3.98	1	5x5	T	-4.963
10	Ne	58	21.565	4.84^{AR}	0	4x4	H	-0.02215
11	Na	166	5.139	0.93	1	5x5	H	-1.643
12	Mg	141	7.646	1.31	2	1x1	V	-1.414
13	Al	121	5.986	1.61	3	5x5	V	-2.863
14	Si	111	8.152	1.90	4	5x5	T	-3.981
15	P	107	10.487	2.19	5	5x5	T	-3.378
16	S	105	10.360	2.58	6	1x1	B	-4.030
17	Cl	102	12.968	3.16	7	5x5	T	-3.116
18	Ar	106	15.760	3.20	0	1x1	H	-0.01677
19	K	203	4.341	0.82	1	5x5	H	-1.950
20	Ca	176	6.113	1.00	2	1x1	H	-2.749
21	Sc	170	6.561	1.36	3	1x1	H	-4.345
22	Ti	160	6.828	1.54	4	1x1	H	-4.428
23	V	153	6.746	1.63	5	1x1	H	-3.857
24	Cr	139	6.767	1.66	6	5x5	H	-2.474
25	Mn	161 (h.s.)	7.434	1.55	7	2x2	H	-2.656
26	Fe	152 (h.s.)	7.902	1.83	7	3x3	H	-3.523
27	Co	150 (h.s.)	7.881	1.88	5	2x2	H	-4.227
28	Ni	124	7.640	1.91	4	2x2	H	-5.132
29	Cu	132	7.726	1.90	4	2x2	H	-2.989
30	Zn	122	9.394	1.65	2	1x1	H	-0.4409
31	Ga	122	5.999	1.81	3	1x1	V	-2.462

32	Ge	120	7.900	2.01	4	2x2	V	-3.493
33	As	119	9.815	2.18	5	2x2	T	-2.907
34	Se	120	9.752	2.55	6	1x1	B	-3.415
35	Br	120	11.814	2.96	7	5x5	T	-2.502
36	Kr	116	14.000	3.00	2	4x4	H	-0.02438
37	Rb	220	4.177	0.82	1	5x5	H	-1.980
38	Sr	195	5.695	0.95	2	1x1	H	-2.432
39	Y	190	6.217	1.22	3	1x1	H	-4.850
40	Zr	175	6.634	1.33	4	1x1	H	-5.778
41	Nb	164	6.759	1.60	5	1x1	H	-5.338
42	Mo	154	7.092	2.16	6	1x1	H	-4.072
43	Tc	147	7.280	1.90	7	4x4	H	-5.206
44	Ru	146	7.361	2.20	8	1x1	V	-5.798
45	Rh	142	7.459	2.28	7	5x5	H	-5.674
46	Pd	139	8.337	2.20	5	4x4	H	-4.293
47	Ag	145	7.576	1.93	3	2x2	H	-1.845
48	Cd	144	8.994	1.69	2	1x1	H	-0.4127
49	In	142	5.786	1.78	3	1x1	V	-2.397
50	Sn	139	7.344	1.96	4	1x1	V	-3.219
51	Sb	139	8.640	2.05	5	2x2	T	-2.552
52	Te	138	9.010	2.10	6	2x2	B	-2.686
53	I	139	10.451	2.66	7	5x5	T	-1.913
54	Xe	140	12.130	2.60	8	2x2	H	-0.01667
55	Cs	244	3.894	0.79	1	5x5	H	-2.142
56	Ba	215	5.212	0.89	2	5x5	H	-2.955
57	La	207	5.577	1.10	3	1x1	H	-5.123
58	Ce	204	5.539	1.12	4	1x1	H	-5.191
59	Pr	203	5.464	1.13	5	1x1	H	-4.949
60	Nd	201	5.525	1.14	4	1x1	H	-4.954
61	Pm	199	5.550	1.07[AR]	3	1x1	H	-4.955
62	Sm	198	5.644	1.17	3	1x1	H	-4.922
63	Eu	198	5.670	1.01[AR]	3	1x1	H	-2.912
64	Gd	196	6.150	1.20	3	1x1	H	-4.940
65	Tb	194	5.864	1.10[AR]	4	1x1	H	-4.830
66	Dy	192	5.939	1.22	4	1x1	H	-4.781
67	Ho	192	6.022	1.23	3	1x1	H	-4.727
68	Er	189	6.108	1.24	3	1x1	H	-4.669
69	Tm	190	6.184	1.25	3	1x1	H	-4.609
70	Yb	187	6.254	1.06[AR]	3	1x1	H	-2.355
71	Lu	187	5.426	1.27	3	1x1	H	-4.506
72	Hf	175	6.825	1.30	4	1x1	H	-5.516
73	Ta	170	7.890	1.50	5	1x1	V	-5.923
74	W	162	7.980	2.36	6	1x1	V	-5.176
75	Re	151	7.880	1.90	7	1x1	V	-5.232
76	Os	144	8.700	2.20	8	1x1	V	-6.697
77	Ir	141	9.100	2.20	9	1x1	V	-6.599
78	Pt	136	9.000	2.28	6	2x2	H	-5.849
79	Au	136	9.226	2.54	5	2x2	H	-2.577
80	Hg	132	10.438	2.00	2	1x1	H	-0.1682
81	Tl	145	6.108	1.62	3	1x1	V	-2.233
82	Pb	146	7.417	2.33	4	1x1	V	-3.087
83	Bi	148	7.289	2.02	5	2x2	T	-2.298
84	Po	140	8.417	2.00	6	2x2	B	-2.295
85	At	150	9.500	2.20	7	5x5	T	-1.664

86	Rn	150	10.745	2.59[M]	6	4x4	H	-0.02922
87	Fr	260	3.900	0.70	1	5x5	H	-1.942
88	Ra	221	5.279	0.90	2	5x5	B	-2.263
89	Ac	215	5.170	1.10	3	1x1	H	-4.395
90	Th	206	6.080	1.30	4	1x1	H	-6.458
91	Pa	200	5.890	1.50	5	1x1	H	-6.248
92	U	196	6.194	1.38	6	1x1	H	-5.407
93	Np	190	6.266	1.36	7	1x1	H	-4.577
94	Pu	187	6.060	1.28	8	1x1	H	-3.777
95	Am	180	5.993	1.30	7	1x1	H	-2.954
96	Cm	169	6.020	1.30	6	1x1	H	-4.025

2. DFT results for all analysed coverages

Tab. S2 below presents the results of DFT calculations for all five investigated coverages, providing adsorption energy (E_a) for every starting configuration. The best configuration for each element (taking into consideration all coverages) are marked green. Best configuration for specific reconstruction is highlighted with bold font style. Additionally, some configurations are marked with “–” symbol, which means that adsorbate atoms do not form such geometry.

Table S2. Adsorption energies calculated by DFT for all analysed coverages.

Atomic nr	Element	1×1				2×2				Element	Atomic nr
		V	T	B	H	V	T	B	H		
1	H	–	**-2.0777**	–	-0.6205	-2.0141	**-2.1569**	–	-2.0140	H	1
2	He	-0.0061	-0.0065	-0.0038	**-0.0082**	-0.0065	-0.0059	-0.0062	**-0.0079**	He	2
3	Li	-1.8984	-1.4183	-1.9133	**-2.0410**	-1.9556	–	–	**-2.1508**	Li	3
4	Be	**-2.3863**	-1.1013	–	–	**-2.4532**	-0.7137	–	-2.0135	Be	4
5	B	**-4.1930**	-2.1331	–	-3.8070	**-4.5313**	-1.8418	-2.7235	-2.9559	B	5
6	C	**-4.5732**	-2.6756	-4.5130	-3.6861	–	**-5.1054**	-4.2867	-3.7474	C	6
7	N	-3.3237	-2.1936	**-3.8704**	-2.2316	-3.5799	-3.4632	**-4.5816**	-3.0931	N	7
8	O	-3.7930	-4.1385	**-5.9140**	-3.0289	–	-4.4828	**-5.7966**	-4.4815	O	8
9	F	–	**-4.6906**	–	-3.0646	–	**-4.8710**	–	-4.8709	F	9
10	Ne	-0.0133	-0.0133	-0.0072	**-0.0151**	-0.0130	-0.0120	-0.0125	**-0.0132**	Ne	10
11	Na	-1.3003	-1.0832	–	**-1.3660**	-1.3024	-1.1627	–	**-1.4704**	Na	11
12	Mg	**-1.4145**	-1.0254	–	-1.3566	-0.9059	-0.3945	-0.3793	**-0.9096**	Mg	12
13	Al	**-2.7817**	-2.4196	–	-2.7501	**-2.8314**	–	–	-2.5676	Al	13
14	Si	**-3.6782**	-2.9503	–	-3.4702	**-3.9654**	–	–	-3.3242	Si	14
15	P	-2.8038	-2.0316	**-2.9380**	-2.6616	-3.2415	**-3.3107**	-2.6390	-2.5652	P	15
16	S	-2.5454	-2.6767	**-4.0301**	-2.5127	-2.9577	-2.8609	**-3.8733**	-2.7336	S	16
17	Cl	–	**-2.8543**	–	-1.9024	-2.8808	**-3.0368**	–	-1.7722	Cl	17
18	Ar	–	-0.0157	-0.0157	**-0.0168**	-0.0111	-0.0102	-0.0100	**-0.0133**	Ar	18
19	K	-1.0330	-0.8349	–	**-1.1491**	-1.4895	-1.3909	–	**-1.6184**	K	19
20	Ca	-2.4157	-1.9039	–	**-2.7495**	-2.1716	–	**-2.2423**	-2.1793	Ca	20
21	Sc	-4.1494	–	–	**-4.3445**	-3.4758	–	-3.6287	**-3.6412**	Sc	21
22	Ti	-4.3525	–	–	**-4.4280**	-3.7293	–	–	**-4.0478**	Ti	22
23	V	-3.6576	–	–	**-3.8573**	-3.1204	–	–	**-3.3184**	V	23
24	Cr	-2.3115	–	–	**-2.3383**	**-1.3083**	–	–	–	Cr	24
25	Mn	**-1.9010**	–	–	–	-1.3697	–	–	**-2.6558**	Mn	25
26	Fe	**-3.2885**	–	–	–	**-2.7573**	–	–	–	Fe	26
27	Co	**-3.9202**	–	–	–	-3.7614	–	–	**-4.2274**	Co	27
28	Ni	**-4.2900**	–	–	–	-4.4836	–	–	**-5.1324**	Ni	28
29	Cu	**-2.4541**	–	–	–	-2.4407	-1.5889	–	**-2.9864**	Cu	29
30	Zn	-0.3508	-0.3580	–	**-0.4409**	-0.0458	-0.2019	–	**-0.2612**	Zn	30

31	Ga	**-2.4617**	-2.1937	–	-2.4385	**-2.4236**	–	–	-2.3574	Ga	31
32	Ge	**-3.3569**	-2.8036	–	-3.0956	**-3.4929**	–	–	-3.0446	Ge	32
33	As	-2.4522	-1.9882	**-2.6130**	-2.2495	-2.8410	**-2.9065**	–	-2.2741	As	33
34	Se	-2.3425	-2.3925	**-3.4153**	-2.3339	-2.7665	-2.4599	**-3.3390**	-2.4597	Se	34
35	Br	–	**-2.2153**	–	-1.4676	-2.2856	**-2.4269**	–	-1.3962	Br	35
36	Kr	–	0.0082	0.0084	**0.0076**	-0.0151	-0.0139	-0.0148	**-0.0173**	Kr	36
37	Rb	-0.8008	-0.6019	–	**-0.9376**	-1.4685	-1.3857	–	**-1.5832**	Rb	37
38	Sr	-2.0134	-1.5563	–	**-2.4319**	-2.0499	–	**-2.1764**	-2.0449	Sr	38
39	Y	-4.5486	–	–	**-4.8503**	-3.7396	–	**-4.1966**	-3.8434	Y	39
40	Zr	-5.6716	–	–	**-5.7776**	-4.5490	–	-4.7671	**-4.9345**	Zr	40
41	Nb	-5.3263	–	–	**-5.3384**	-4.4113	–	-4.3103	**-4.6196**	Nb	41
42	Mo	-3.9468	–	–	**-4.0720**	**-3.5139**	–	–	-3.4402	Mo	42
43	Tc	**-5.1832**	–	–	–	-4.5816	–	-4.6561	**-4.6891**	Tc	43
44	Ru	**-5.7977**	–	–	–	-5.2779	–	–	**-5.3206**	Ru	44
45	Rh	**-5.4744**	–	–	–	-5.3224	–	–	**-5.5066**	Rh	45
46	Pd	**-3.4649**	–	–	–	-3.7567	–	–	**-4.2643**	Pd	46
47	Ag	-1.7811	-1.5273	–	**-1.8396**	-1.4986	-1.1499	–	**-1.8445**	Ag	47
48	Cd	-0.3347	-0.4025	–	**-0.4127**	-0.0489	-0.1735	–	**-0.1836**	Cd	48
49	In	**-2.3971**	-2.1693	–	-2.2974	-2.1622	–	–	**-2.1853**	In	49
50	Sn	**-3.2189**	-2.8332	–	-2.8192	**-3.1032**	–	–	-2.8895	Sn	50
51	Sb	-2.3239	-2.1752	**-2.3936**	-1.9269	-2.5099	**-2.5523**	-2.5143	-2.0962	Sb	51
52	Te	-1.9475	-2.2411	**-2.5269**	-1.8159	-2.4732	-2.2393	**-2.6863**	-2.0036	Te	52
53	I	-0.8870	**-1.5342**	-1.4710	-0.9638	–	**-1.8467**	–	-1.0048	I	53
54	Xe	–	0.1449	0.1478	**0.1429**	-0.0043	-0.0051	0.0021	**-0.0167**	Xe	54
55	Cs	-0.6274	-0.3484	–	**-0.8402**	-1.6118	–	–	**-1.7138**	Cs	55
56	Ba	-2.2365	–	–	**-2.6211**	-2.6867	–	**-2.8511**	-2.7443	Ba	56
57	La	-4.8271	–	–	**-5.1225**	-4.3028	–	**-4.8366**	-4.5721	La	57
58	Ce	-4.9006	–	–	**-5.1913**	–	-4.1642	**-4.7038**	-4.4140	Ce	58
59	Pr	-4.6725	–	–	**-4.9488**	-4.0283	–	**-4.5440**	-4.3045	Pr	59
60	Nd	-4.6591	–	–	**-4.9544**	-3.9901	–	**-4.4946**	-4.2573	Nd	60
61	Pm	-4.6446	–	–	**-4.9551**	-3.9523	–	**-4.4468**	-4.2276	Pm	61
62	Sm	-4.6047	–	–	**-4.9225**	-3.8888	–	**-4.3725**	-4.1577	Sm	62
63	Eu	-2.4422	-1.8943	–	**-2.9118**	-2.3931	–	**-2.5094**	-2.4585	Eu	63
64	Gd	-4.6190	–	–	**-4.9404**	-3.8555	–	**-4.3158**	-4.1858	Gd	64

65	Tb	-4.5145	–	–	**-4.8303**	-3.7284	–	**-4.1727**	-3.8179	Tb	65
66	Dy	-4.4744	–	–	**-4.7809**	-3.6697	–	**-4.0930**	-3.7480	Dy	66
67	Ho	-4.4336	–	–	**-4.7275**	-3.6140	–	**-4.0130**	-3.6784	Ho	67
68	Er	-4.3893	–	–	**-4.6685**	-3.5603	–	**-3.9330**	-3.6234	Er	68
69	Tm	-4.3456	–	–	**-4.6088**	-3.5109	–	**-3.8534**	-3.5582	Tm	69
70	Yb	-1.9592	–	–	**-2.3547**	-1.6972	-0.6310	**-1.7297**	-1.7033	Yb	70
71	Lu	-4.2787	–	–	**-4.5057**	-3.4470	-1.9738	**-3.7149**	-3.4532	Lu	71
72	Hf	-5.4739	–	–	**-5.5162**	-4.1998	–	-4.4715	**-4.6118**	Hf	72
73	Ta	**-5.9227**	–	–	-5.8266	-4.9093	–	-4.9248	**-5.1788**	Ta	73
74	W	**-5.1762**	–	–	-5.1629	**-4.7421**	–	-4.5848	-4.6281	W	74
75	Re	**-5.2323**	–	–	–	**-4.6061**	-4.4731	–	-4.5981	Re	75
76	Os	**-6.6965**	–	–	–	-5.6256	–	**-6.1759**	-5.8402	Os	76
77	Ir	**-6.5986**	–	–	–	–	-6.0516	–	**-6.3102**	Ir	77
78	Pt	-5.1946	–	–	**-5.4177**	-5.1824	–	–	**-5.8488**	Pt	78
79	Au	-2.3123	-2.1923	-2.3762	**-2.5178**	-2.1803	-1.9814	-2.1391	**-2.5765**	Au	79
80	Hg	-0.0977	-0.1516	-0.1515	**-0.1682**	-0.0414	-0.0969	-0.0918	**-0.1063**	Hg	80
81	Tl	**-2.2328**	-1.9898	–	-2.1019	-1.9943	–	–	**-2.0787**	Tl	81
82	Pb	**-3.0871**	-2.7530	–	-2.6840	**-2.8544**	–	–	-2.7750	Pb	82
83	Bi	-2.2356	-2.2226	**-2.2660**	-1.8832	-2.2564	**-2.2979**	-2.2660	-1.9140	Bi	83
84	Po	-1.7456	**-2.1452**	-2.1028	-1.6233	-2.2065	-1.9358	**-2.2947**	-2.2655	Po	84
85	At	-0.9986	**-1.3310**	–	-1.0317	-1.4970	**-1.6017**	–	-0.8708	At	85
86	Rn	–	0.2221	0.2217	**0.2201**	-0.0191	-0.0189	-0.0197	**-0.0234**	Rn	86
87	Fr	-0.4161	-0.1653	–	**-0.6088**	-1.4143	-1.3386	–	**-1.5081**	Fr	87
88	Ra	-1.4657	-1.0676	–	**-1.8038**	-2.0040	–	**-2.1402**	-1.9847	Ra	88
89	Ac	-4.1825	–	–	**-4.3949**	-3.5987	–	**-4.2051**	-3.6610	Ac	89
90	Th	-6.4075	–	–	**-6.4581**	-4.7934	–	**-5.6012**	-5.1750	Th	90
91	Pa	-6.1616	–	–	**-6.2484**	-4.2900	-4.0967	**-5.3197**	-4.9005	Pa	91
92	U	-5.1516	–	–	**-5.4073**	-3.7433	-4.5002	**-4.5003**	-4.2623	U	92
93	Np	-4.2922	–	–	**-4.5769**	-3.1603	–	**-3.6538**	-2.5246	Np	93
94	Pu	-3.4491	–	–	**-3.7692**	0.0155	-0.4794	-0.5016	**-0.5621**	Pu	94
95	Am	-2.5896	–	–	**-2.9661**	**-2.1075**	2.0456	1.7229	1.8534	Am	95
96	Cm	0.5904	–	–	**0.2564**	1.1941	–	0.8739	**-3.0663**	Cm	96

Atomic nr	Element	3×3 V	3×3 T	3×3 B	3×3 H	4×4 V	4×4 T	4×4 B	4×4 H	Element	Atomic nr
1	H	-2.0124	**-2.1024**	–	-0.4245	-2.0434	**-2.1159**	–	-2.1056	H	1
2	He	-0.0082	-0.0079	-0.0074	**-0.0097**	-0.0083	-0.0082	-0.0075	**-0.0087**	He	2
3	Li	-1.9600	-1.7760	–	**-2.2000**	-2.0028	-1.8307	–	**-2.2371**	Li	3
4	Be	**-2.4041**	-0.6856	–	-1.6333	**-2.4154**	-0.6873	–	-1.5954	Be	4
5	B	**-4.6903**	-1.7796	-2.6354	-2.8063	**-4.7992**	-1.8187	-2.6345	-2.8626	B	5
6	C	-5.4571	**-5.5611**	-4.2422	-3.7942	-5.5935	**-5.7091**	-4.2972	-3.9620	C	6
7	N	-3.6215	-2.0644	**-4.5931**	-3.2322	-3.7002	-1.7197	**-4.6638**	-3.4544	N	7
8	O	-4.3877	-4.4648	**-5.7839**	-4.4593	-4.4202	-4.5083	**-5.7868**	-4.4984	O	8
9	F	–	**-4.8989**	–	-4.8986	–	**-4.9434**	–	-4.9411	F	9
10	Ne	-0.0176	-0.0168	-0.0164	**-0.0187**	-0.0209	-0.0202	-0.0203	**-0.0221**	Ne	10
11	Na	-1.4131	-1.2857	–	**-1.5613**	-1.4770	-1.3347	–	**-1.6163**	Na	11
12	Mg	**-0.8706**	-0.4083	-0.3973	-0.7506	**-0.8619**	-0.4120	-0.4063	-0.7090	Mg	12
13	Al	**-2.8088**	-2.7215	–	-2.5265	**-2.8445**	-2.7371	–	-2.5212	Al	13
14	Si	**-3.9641**	–	–	-3.0287	–	**-3.9608**	–	-3.0109	Si	14
15	P	-3.2299	**-3.2998**	-2.6090	-3.2297	-3.2600	**-3.3427**	-2.6427	-2.2628	P	15
16	S	–	-2.7811	**-3.8571**	-3.8564	–	-2.8186	**-3.8646**	-2.4160	S	16
17	Cl	–	**-3.0483**	–	-1.8054	–	**-3.0926**	–	-1.8838	Cl	17
18	Ar	-0.0127	-0.0119	-0.0128	**-0.0155**	-0.0130	-0.0126	-0.0124	**-0.0148**	Ar	18
19	K	-1.7183	-1.6289	–	**-1.8265**	-1.8123	-1.7128	-1.7804	**-1.9127**	K	19
20	Ca	-2.1137	-2.0328	-2.1851	**-2.2073**	-2.1572	-2.0939	-2.2087	**-2.2165**	Ca	20
21	Sc	-3.4758	–	**-3.6941**	-3.5650	-3.4945	-3.3194	**-3.7528**	-3.5326	Sc	21
22	Ti	-3.7095	–	–	**-3.8486**	**-3.7060**	-3.5204	–	-3.5374	Ti	22
23	V	-3.2616	–	–	**-3.4985**	-3.2709	-3.1002	–	**-3.5167**	V	23
24	Cr	-1.1687	-2.1741	–	**-2.4589**	-1.1736	-1.9478	–	**-2.4668**	Cr	24
25	Mn	-1.1421	–	-1.0724	**-2.5830**	-1.0966	-1.0421	–	**-2.5643**	Mn	25
26	Fe	-3.2679	–	–	**-3.5229**	-3.2572	-3.1208	–	**-3.5199**	Fe	26
27	Co	-3.6856	–	–	**-4.1016**	-3.6673	-3.5299	–	**-4.1205**	Co	27
28	Ni	-4.4894	–	–	**-5.0914**	-4.4844	-4.2861	–	**-5.0893**	Ni	28
29	Cu	-2.3904	-1.5618	–	**-2.7234**	-2.3908	-1.5685	–	**-2.7343**	Cu	29
30	Zn	-0.0471	-0.2072	–	**-0.2506**	-0.0472	-0.2054	-0.1973	**-0.2613**	Zn	30
31	Ga	**-2.3783**	-2.2583	–	-2.3401	**-2.4179**	-2.2770	–	-2.3415	Ga	31
32	Ge	**-3.4229**	-3.3969	–	-2.7002	**-3.4084**	-3.3817	–	-2.6615	Ge	32

33	As	-2.7873	**-2.8281**	–	-2.7880	-2.8030	**-2.8520**	–	-2.8029	As	33
34	Se	-2.6164	-2.3438	**-3.3155**	-3.3154	-2.6558	-2.3779	**-3.3255**	-2.0882	Se	34
35	Br	–	**-2.4353**	–	-1.4203	–	**-2.4792**	–	-1.5007	Br	35
36	Kr	-0.0195	-0.0180	-0.0190	**-0.0204**	-0.0220	-0.0220	-0.0227	**-0.0244**	Kr	36
37	Rb	-1.7385	-1.6639	-1.7318	**-1.8304**	-1.8477	-1.7675	-1.8232	**-1.9320**	Rb	37
38	Sr	-2.0544	-2.0448	**-2.1637**	-2.1438	-2.1171	-2.1324	**-2.1947**	-2.1804	Sr	38
39	Y	-3.7458	–	**-4.2179**	-3.8906	-3.7748	-3.6217	**-4.2459**	-3.8683	Y	39
40	Zr	-4.5710	–	**-4.8571**	-4.7654	-4.5670	–	**-4.9201**	-4.7443	Zr	40
41	Nb	-4.3628	–	–	**-4.5063**	-4.3517	-4.2082	–	**-4.4293**	Nb	41
42	Mo	**-3.3746**	–	–	-3.2204	**-3.3857**	-3.2843	–	-3.1345	Mo	42
43	Tc	-4.4259	–	–	**-4.5370**	-4.4051	-4.3680	–	**-4.5560**	Tc	43
44	Ru	-5.2034	–	–	**-5.2342**	-5.1884	-5.1098	–	**-5.2178**	Ru	44
45	Rh	-5.3072	–	–	**-5.5791**	-5.3090	-5.1701	–	**-5.6560**	Rh	45
46	Pd	-3.7788	–	–	**-4.2875**	-3.7833	-3.5716	–	**-4.2933**	Pd	46
47	Ag	-1.4752	-1.1280	–	**-1.7594**	-1.4749	-1.1448	–	**-1.7660**	Ag	47
48	Cd	-0.0494	-0.1789	-0.1764	**-0.1930**	-0.0498	-0.1754	-0.1627	**-0.1948**	Cd	48
49	In	-2.0999	-1.9201	–	**-2.1764**	-2.1378	-1.9520	–	**-2.1867**	In	49
50	Sn	**-2.9911**	-2.9121	–	-2.5416	**-2.9706**	-2.9006	–	-2.5013	Sn	50
51	Sb	**-2.4274**	-2.4106	–	-1.6872	**-2.4331**	-2.4142	–	-1.6663	Sb	51
52	Te	-2.3167	-1.8103	**-2.6508**	-2.4531	-2.3411	-1.8204	**-2.6566**	-2.4505	Te	52
53	I	–	**-1.8463**	–	-1.0219	–	**-1.8868**	–	-1.1032	I	53
54	Xe	-0.0049	0.0022	-0.0013	**-0.0105**	-0.0091	-0.0019	-0.0054	**-0.0107**	Xe	54
55	Cs	-1.9005	-1.8381	-1.8783	**-1.9831**	-2.0195	-1.9542	-2.0020	**-2.0921**	Cs	55
56	Ba	-2.7391	-2.7720	-2.8444	**-2.8731**	-2.8130	-2.8685	-2.8762	**-2.9167**	Ba	56
57	La	-4.2896	-4.1280	**-4.8282**	-4.5623	-4.3135	-4.1717	**-4.8237**	-4.5438	La	57
58	Ce	-4.1772	-4.5067	**-4.7292**	-4.4028	-4.1962	-3.9061	**-4.7154**	-4.3885	Ce	58
59	Pr	-3.9936	-3.8437	**-4.5355**	-4.2437	-4.0161	-3.8779	**-4.5429**	-4.2228	Pr	59
60	Nd	-3.9602	-3.8064	**-4.4897**	-4.1937	-3.9768	-3.8352	**-4.4977**	-4.1685	Nd	60
61	Pm	-3.9308	-3.7791	**-4.4510**	-4.1485	-3.9559	-3.8041	**-4.4670**	-4.1278	Pm	61
62	Sm	-3.8772	-3.7242	**-4.3814**	-4.0702	-3.9003	-3.7434	**-4.4005**	-4.0485	Sm	62
63	Eu	-2.3664	-2.3073	**-2.5131**	-2.4918	-2.4077	-2.3874	**-2.5334**	-2.5089	Eu	63
64	Gd	-3.8574	-3.7103	**-4.3355**	-4.0032	-3.8857	-3.7309	**-4.3614**	-3.9801	Gd	64
65	Tb	-3.7354	-3.5915	**-4.1966**	-3.8518	-3.7645	-3.6128	**-4.2254**	-3.8264	Tb	65
66	Dy	-3.6784	–	**-4.1208**	-3.7627	-3.7076	-3.5594	**-4.1521**	-3.7383	Dy	66

67	Ho	-3.6224	-3.4855	**-4.0441**	-3.6721	-3.6553	-3.5089	**-4.0764**	-3.6455	Ho	67
68	Er	-3.5676	-3.4347	**-3.9661**	-3.5819	-3.5997	-3.4569	**-4.0032**	-3.5554	Er	68
69	Tm	-3.5174	-3.3865	**-3.8926**	-3.4897	-3.5509	-3.4065	**-3.9293**	-3.4620	Tm	69
70	Yb	**-1.6697**	-0.6733	-1.6615	-1.6517	**-1.6990**	-0.7147	-1.6918	-1.6880	Yb	70
71	Lu	-3.4549	-1.9867	**-3.7595**	-3.3272	-3.4922	-2.0380	**-3.8000**	-3.2955	Lu	71
72	Hf	-4.2234	-4.0837	–	**-4.2644**	-4.2035	-4.0330	–	**-4.3754**	Hf	72
73	Ta	-4.8256	-4.6937	–	**-4.8466**	**-4.8011**	-4.6935	–	-4.7704	Ta	73
74	W	**-4.5401**	–	–	-4.1860	**-4.5504**	-4.4902	–	-4.0982	W	74
75	Re	-4.3544	-4.3967	**-4.8388**	-4.0432	**-4.3462**	–	–	-3.9276	Re	75
76	Os	-5.4844	–	–	**-5.5529**	**-5.4704**	-5.4523	–	-5.4034	Os	76
77	Ir	-5.9596	–	–	**-6.2748**	-5.9453	-5.8826	–	**-6.3541**	Ir	77
78	Pt	-5.1779	–	–	**-5.8131**	-5.1868	-5.0578	-4.9865	**-5.8175**	Pt	78
79	Au	-2.2064	-1.9468	-2.0625	**-2.4322**	-1.8521	-1.9730	-2.0826	**-2.4059**	Au	79
80	Hg	-0.0458	-0.1026	-0.0988	**-0.1126**	-0.0498	-0.1049	-0.0714	**-0.1165**	Hg	80
81	Tl	-1.9468	-1.7466	-1.9452	**-2.0911**	-1.9878	-1.7687	–	**-2.1159**	Tl	81
82	Pb	**-2.7214**	-2.6085	–	-2.4088	**-2.7036**	-2.6184	–	-2.3631	Pb	82
83	Bi	**-2.1630**	-2.1198	–	-1.7505	**-2.1622**	-2.1184	–	-1.4839	Bi	83
84	Po	-2.0531	-1.7865	**-2.2541**	-2.1401	-2.0787	-1.5029	**-2.2628**	-2.1451	Po	84
85	At	-1.5063	**-1.5975**	–	-0.8684	-1.5370	**-1.6358**	-1.6277	-0.9505	At	85
86	Rn	-0.0227	-0.0240	-0.0237	**-0.0254**	-0.0269	-0.0280	-0.0273	**-0.0292**	Rn	86
87	Fr	-1.7041	-1.6461	-1.6856	**-1.7789**	-1.8203	-1.7674	-1.8049	**-1.8900**	Fr	87
88	Ra	-2.0756	-2.1377	**-2.1614**	-2.1458	-2.1533	**-2.2405**	-2.2107	-2.2068	Ra	88
89	Ac	-3.6166	-3.5257	**-4.1810**	-3.7458	-3.6427	-3.5441	**-4.1718**	-3.7300	Ac	89
90	Th	-4.7984	-4.6864	**-5.5791**	-5.1375	-4.7785	-4.5890	**-5.5712**	-5.2883	Th	90
91	Pa	-4.3482	-4.0444	**-5.2056**	-4.7549	-4.3385	–	**-5.2416**	-4.6913	Pa	91
92	U	-3.1677	–	**-4.0289**	-3.5818	-3.7545	-3.5992	**-4.0169**	-3.5413	U	92
93	Np	-1.7772	-2.0996	**-2.4701**	-2.1450	-1.7820	–	**-2.4516**	-2.0783	Np	93
94	Pu	-0.0309	0.1315	**-0.5885**	-0.2463	-0.0617	**-2.5445**	-0.5777	-0.2511	Pu	94
95	Am	**-2.0894**	2.1841	1.6464	1.8742	1.9799	2.1602	**1.6972**	1.8671	Am	95
96	Cm	-3.0723	-2.8992	**-3.3555**	1.0951	-3.0931	-2.9310	**-3.3636**	-2.9988	Cm	96

Atomic nr	Element	5×5 V	5×5 T	5×5 B	5×5 H
1	H	-2.0727	-2.1074	-2.1286	**-2.1355**
2	He	-0.0006	-0.0001	0.0003	**-0.0018**
3	Li	-2.0203	-1.8389	-2.0116	**-2.2547**
4	Be	**-2.4091**	-0.6918	-2.4074	-1.6133
5	B	**-4.8465**	-1.8026	-2.6479	-2.9154
6	C	-5.6816	**-5.7970**	-4.3018	-4.0216
7	N	-3.8325	-1.6993	**-4.6795**	-3.5431
8	O	-4.4329	-4.5321	**-5.7786**	-5.0712
9	F	-4.7923	**-4.9632**	–	-4.9594
10	Ne	-0.0179	-0.0166	-0.0175	**-0.0195**
11	Na	-1.4997	-1.3605	-1.4800	**-1.6432**
12	Mg	**-0.8611**	-0.4087	-0.3995	-0.7208
13	Al	**-2.8628**	-1.9116	–	-2.5361
14	Si	–	**-3.9809**	–	-3.0352
15	P	-3.3073	**-3.3780**	-2.6561	-2.3006
16	S	–	-2.8193	**-3.8590**	-2.4663
17	Cl	–	**-3.1162**	–	-1.9025
18	Ar	-0.0058	-0.0047	-0.0049	**-0.0069**
19	K	-1.8346	-1.7667	-1.8261	**-1.9505**
20	Ca	-2.1502	-2.1001	-2.1922	**-2.2405**
21	Sc	-3.5269	-3.3482	**-3.7637**	-3.5442
22	Ti	**-3.7082**	-3.5217	–	-3.5360
23	V	-3.2661	-3.1029	-3.2814	**-3.5351**
24	Cr	-1.1716	-1.9269	–	**-2.4736**
25	Mn	-1.0746	-1.0183	–	**-1.4259**
26	Fe	**-3.2671**	-3.1300	–	-3.0027
27	Co	-3.6719	-3.5276	–	**-4.0518**
28	Ni	-4.4800	-4.2928	–	**-5.0788**
29	Cu	-2.3935	-1.5755	–	**-2.7543**
30	Zn	-0.0400	-0.1992	-0.1827	**-0.2526**
31	Ga	**-2.4244**	-1.8696	–	-2.3491
32	Ge	**-3.4204**	-3.3872	–	-2.6770

33	As	-2.8402	**-2.8715**	–	-1.9530
34	Se	-3.3183	-2.3496	**-3.3211**	-2.1325
35	Br	–	**-2.5021**	–	-1.5214
36	Kr	-0.0208	-0.0193	-0.0191	**-0.0216**
37	Rb	-1.8955	-1.8157	-1.8570	**-1.9797**
38	Sr	-2.1207	-2.1663	-2.1845	**-2.2225**
39	Y	-3.8098	-3.6551	**-4.2513**	-3.8856
40	Zr	-4.5750	-4.3799	**-4.9362**	-4.7502
41	Nb	-4.3664	-4.2314	–	**-4.4210**
42	Mo	**-3.3850**	-3.2868	–	-3.1375
43	Tc	-4.4190	-4.3742	–	**-4.5871**
44	Ru	-5.2033	-5.1134	–	**-5.2455**
45	Rh	-5.3448	-5.1934	–	**-5.6743**
46	Pd	-3.7799	-3.5602	–	**-4.2741**
47	Ag	-1.4831	-1.1505	-1.3321	**-1.7862**
48	Cd	-0.0424	-0.1713	-0.1568	**-0.1878**
49	In	-2.1376	-1.8034	–	**-2.1974**
50	Sn	**-2.9837**	-2.9054	–	-2.5124
51	Sb	**-2.4643**	-2.4205	–	-1.6927
52	Te	-2.3402	-1.8036	**-2.6392**	-2.0240
53	I	–	**-1.9129**	–	-1.1259
54	Xe	-0.0011	-0.0046	-0.0034	**-0.0087**
55	Cs	-2.0641	-2.0123	-2.0551	**-2.1423**
56	Ba	-2.8284	-2.9085	-2.9238	**-2.9553**
57	La	-4.3248	-4.1552	**-4.8289**	-4.5635
58	Ce	-4.2011	-3.9058	**-4.7264**	-4.4034
59	Pr	-4.0280	-3.8488	**-4.5475**	-4.2407
60	Nd	-3.9973	-3.8087	**-4.4975**	-4.1818
61	Pm	-3.9794	-3.8206	**-4.4729**	-4.1447
62	Sm	-3.9317	-3.7620	**-4.4070**	-4.0660
63	Eu	-2.4184	-2.4147	-2.5257	**-2.5380**
64	Gd	-3.9202	-3.7575	**-4.3657**	-3.9979
65	Tb	-3.7992	-3.6428	**-4.2317**	-3.8460
66	Dy	-3.7405	-3.5919	**-4.1586**	-3.7558

67	Ho	-3.6942	-1.7575	**-4.0846**	-3.6651
68	Er	-3.6379	-1.8588	**-4.0102**	-3.5725
69	Tm	-3.5926	-1.8577	**-3.9371**	-3.4792
70	Yb	-1.7018	-0.7522	**-1.7128**	-1.6942
71	Lu	-3.5272	-2.0626	**-3.8078**	-3.3140
72	Hf	-4.2276	-4.0648	–	**-4.3718**
73	Ta	**-4.8128**	-4.7161	–	-4.7398
74	W	**-4.5393**	-4.4869	–	-4.0892
75	Re	-4.3519	-4.4022	**-5.2081**	-3.9576
76	Os	-5.4843	-5.4449	**-6.4325**	-5.4377
77	Ir	-5.9773	-5.8875	–	**-6.3695**
78	Pt	-5.1811	-5.0464	–	**-5.8153**
79	Au	-1.8766	-1.9943	-2.0945	**-2.4271**
80	Hg	-0.0469	-0.1035	-0.0675	**-0.1140**
81	Tl	-1.9983	-1.7815	-1.9967	**-2.1353**
82	Pb	**-2.7106**	-2.6100	–	-2.3716
83	Bi	**-2.1863**	-2.1054	–	-1.5276
84	Po	-2.0809	-1.4851	**-2.2265**	-2.1385
85	At	-1.5566	**-1.6636**	-1.6508	-0.9735
86	Rn	-0.0248	-0.0253	-0.0248	**-0.0269**
87	Fr	-1.8576	-1.8142	-1.8433	**-1.9420**
88	Ra	-2.1768	-2.2764	**-2.2765**	-2.2568
89	Ac	-3.6745	-3.5494	**-4.1710**	-3.7479
90	Th	-4.8182	-4.5953	**-5.5926**	-5.2888
91	Pa	-4.3623	-4.0335	**-5.2728**	-4.6833
92	U	-3.7928	-2.9770	**-4.0606**	-3.5426
93	Np	-1.8169	-1.5998	**-2.4645**	-2.0284
94	Pu	-0.0888	0.1169	**-0.5989**	-0.2235
95	Am	1.9647	2.1684	1.6618	**-2.1492**
96	Cm	**-3.1387**	1.2786	0.8397	-3.0104

3. Graphical representation of DFT results in the form of periodic tables

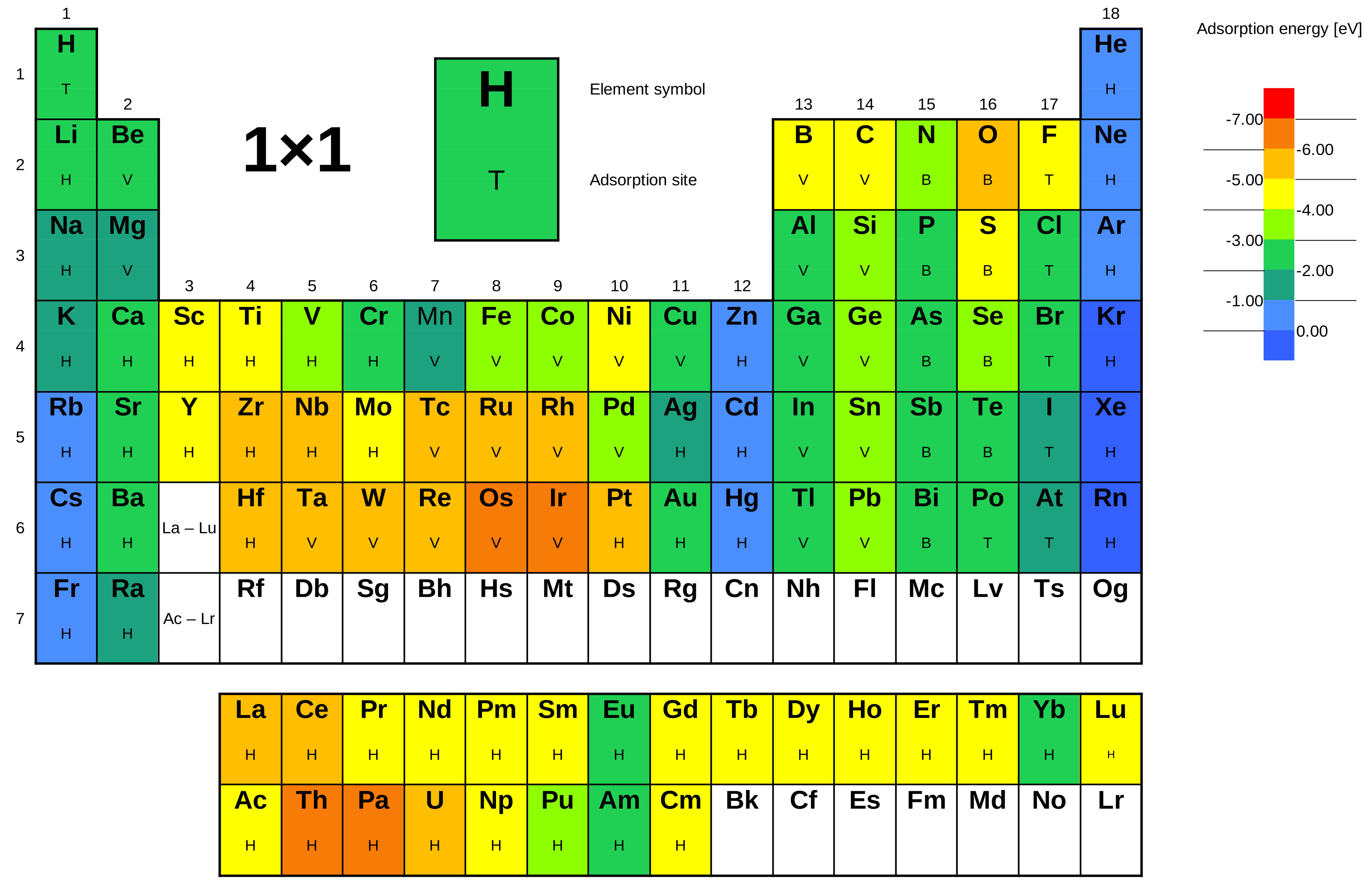

Figure S1. Summary of DFT results for 1×1 coverage.

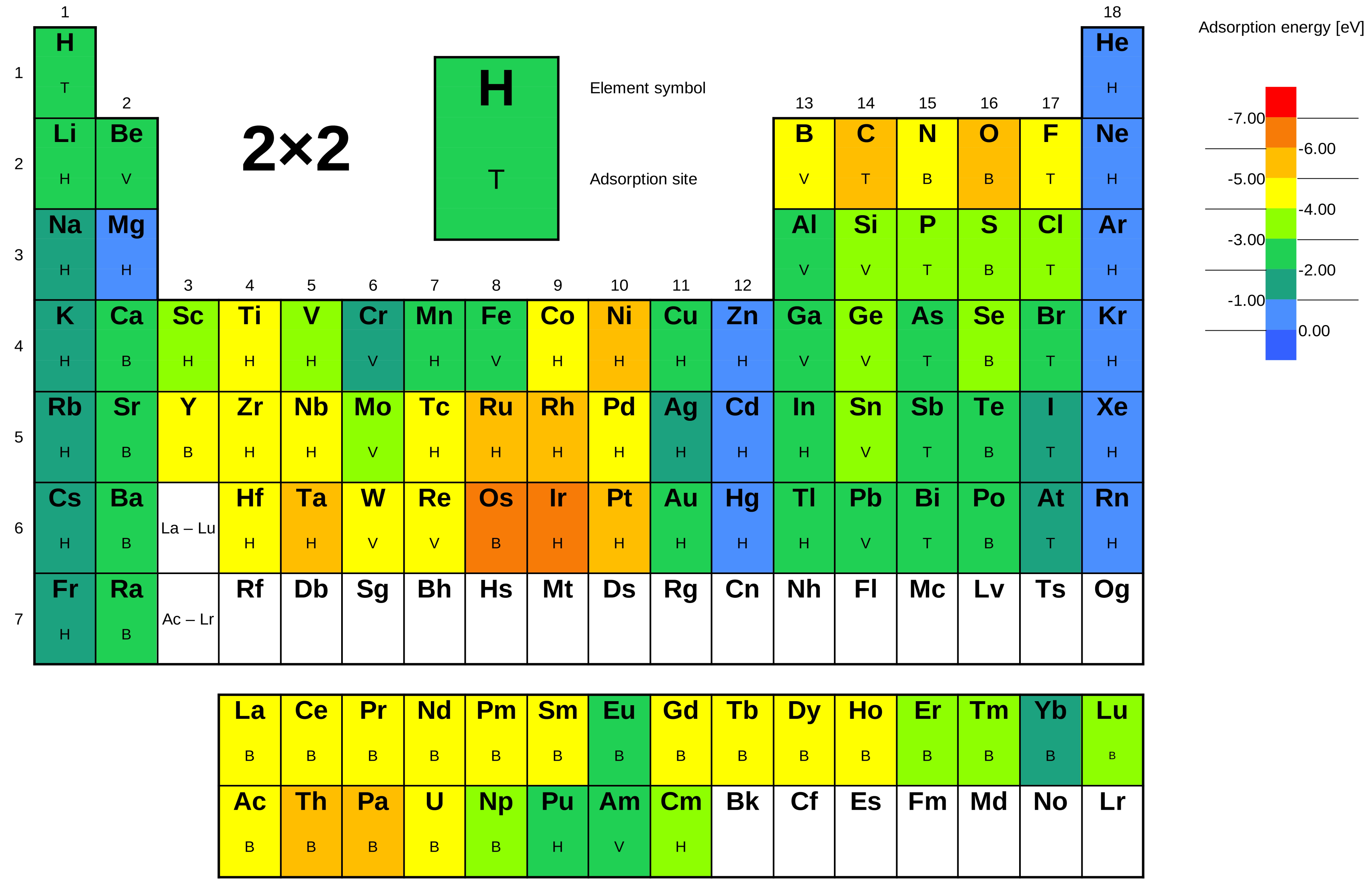

Figure S2. Summary of DFT results for 2×2 coverage.

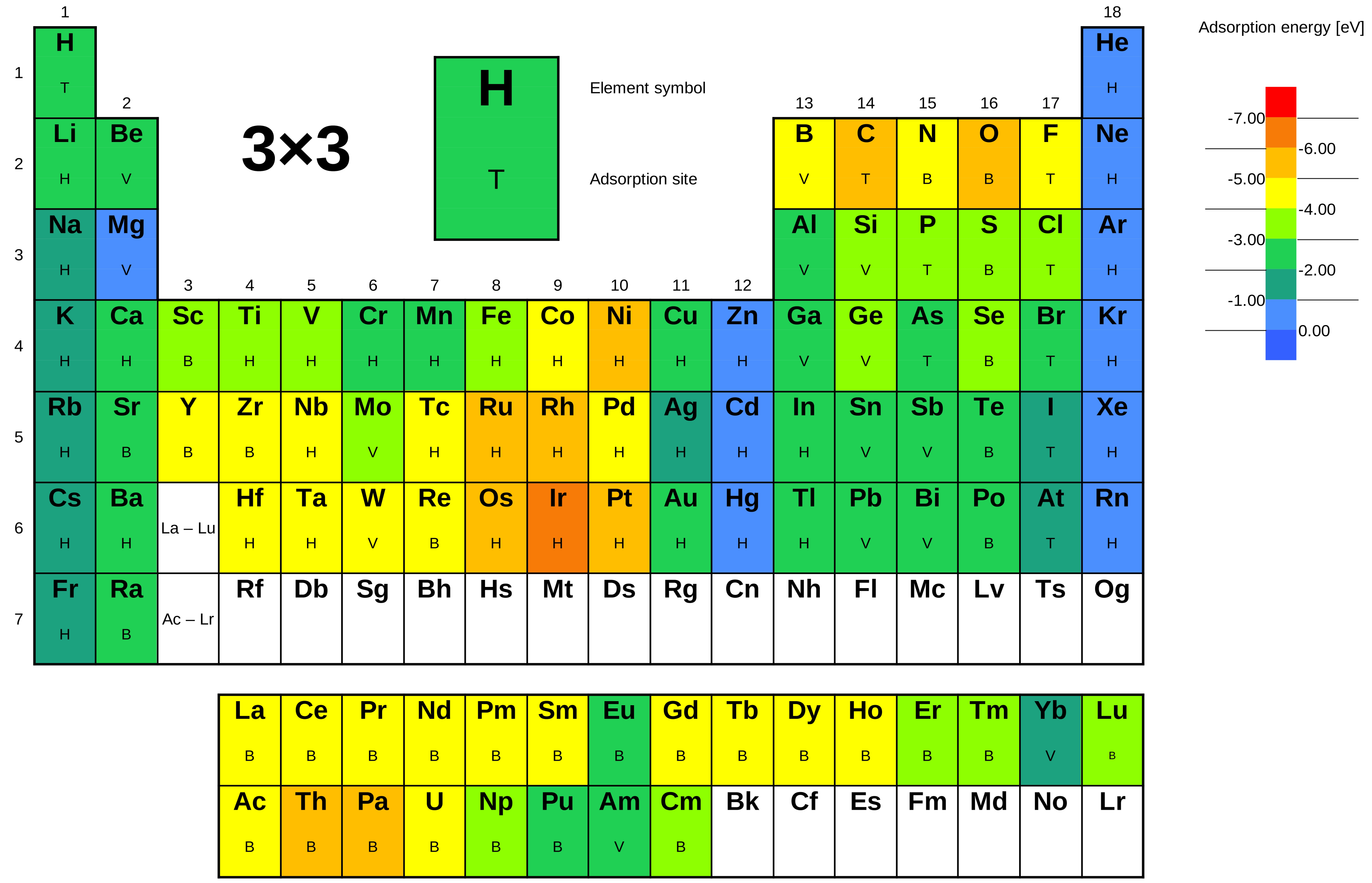

Figure S3. Summary of DFT results for 3×3 coverage.

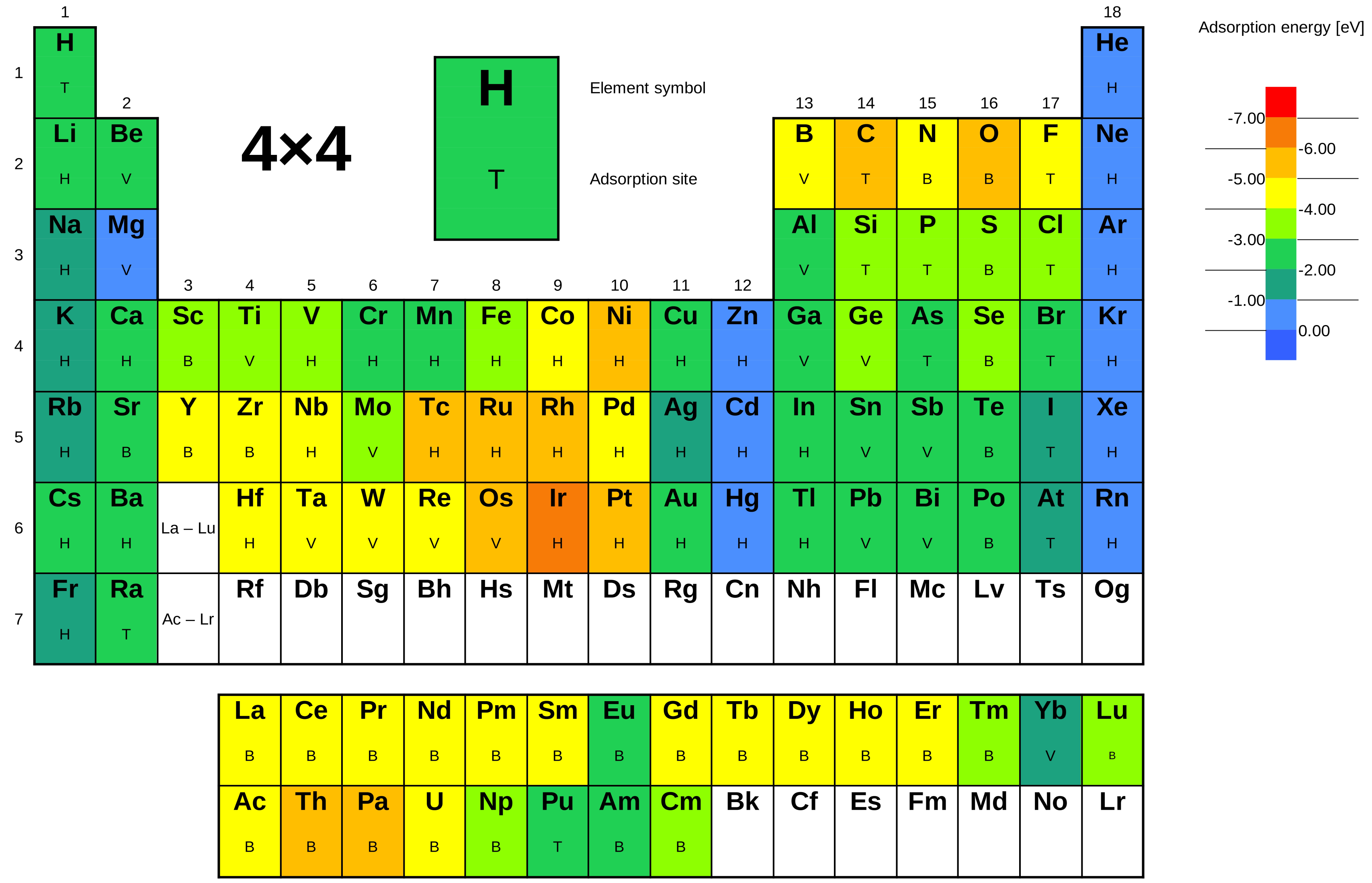

Figure S4. Summary of DFT results for 4×4 coverage.

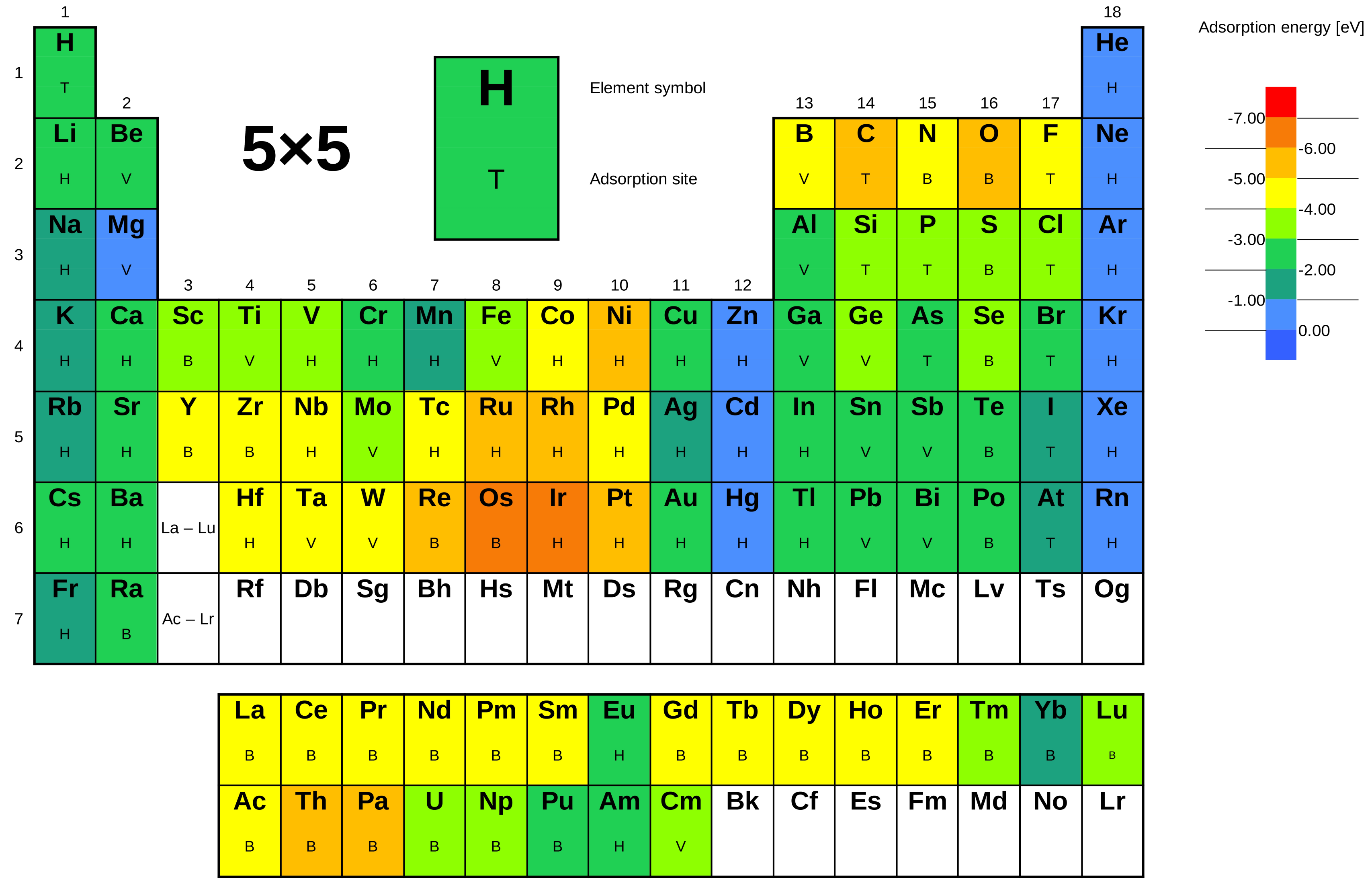

Figure S5. Summary of DFT results for 5×5 coverage.

4. Error matrix, definitions of used statistical metrics and their equations

For the adsorption geometry description, four distinct classes has been defined, each oriented towards one adsorption site. For H class error matrix would look as follows:

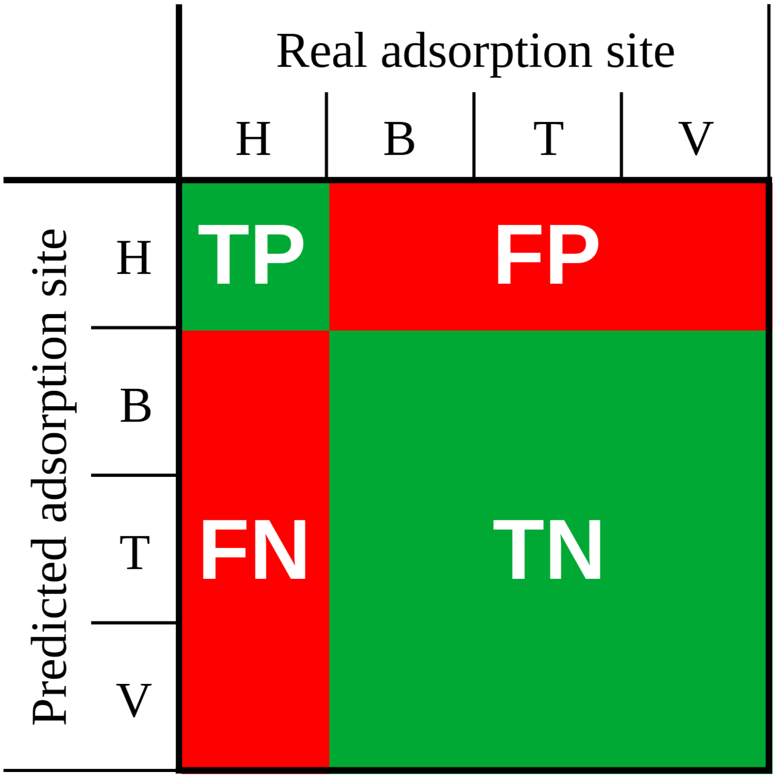

Figure S6. Textbook error matrix in described problem.

where TP means *true positive* (model recognized H geometry as H), TN means *true negative* (model recognized non-H geometry as non-H), FP means *false positive* (model recognized non-H geometry as H) and FN means *false negative* (model recognized H geometry as non-H). Error matrices for other classes look similarly. The number of answers is summed for calculation of statistical metrics.

Accuracy (ACC) shows how many predictions out of all attempts were correct (i.e. overall proportion of correct predictions made by the model):

$$ACC = \frac{T_H + T_B + T_T + T_V}{total}$$

where T_H means number of instances where H class was properly identified as H etc.

Precision (PPV – positive predictive value) measures, among all the times the model predicted a specific adsorption site (e.g. “H” site), how often it was correct.

$$PPV = \frac{TP}{TP + FP}$$

Recall (TPR – true positive rate) shows, among all the adsorption systems that *actually* belonged to a certain site (e.g. “H” site), how many the model correctly identified.

$$TPR = \frac{TP}{TP + FN}$$

F1 Score is the harmonic mean of precision and recall, providing a single score that balances both of them.

$$F1 = 2 \cdot \frac{PPV \cdot TPR}{PPV + TPR}$$

Receiver Operating Characteristic (ROC) curve is plotted by calculating the TPR and *False Positive Rate* (FPR) for different decision threshold (i.e. above which probability model counts item as positive).

$$FPR = \frac{FP}{FP + TN}$$

Area under the receiver operating characteristic curve (ROC AUC) is, as the name suggests, the area under the above-mentioned curve. ROC AUC describes the performance of the model in distinction between positive and negative cases – value approaching 1.0 indicates that the model is excellent at distinguishing between the different possible adsorption geometries.

Macro-average presents the average statistical metric across all classes, with each class given equal weight. It is derived by calculating the statistical metric for each class separately and performing an

average. When X is desired statistical metrics and N the number of classes, the macro average is as follows:

$$\overline{X_{macro}} = \frac{X_{class\ A} + X_{class\ B} + \ldots + X_{class\ N}}{N}$$

Micro-average presents the average statistical metric across entire dataset, with each instance given equal weight regardless of class. It is obtained by totalling the TP, TN, FP and FN predictions and then calculating the statistic metrics in this chapter. The Tab. 2 in the article with micro-averaged metrics would look as follows:

Table S3. Classification micro-average metrics for ML models, predicting the adsorption site.

	**ACC**	**PPV**	**TPR**	**F1**	**ROC AUC**
**ANN**	0.9667	0.9667	0.9667	0.9667	0.9949
**RF**	0.8333	0.8333	0.8333	0.8333	0.9552
**GBT**	0.8167	0.8167	0.8167	0.8167	0.9410
**LGBM**	0.7583	0.7833	0.7833	0.7833	0.9331
**XBM**	0.7833	0.7583	0.7583	0.7583	0.9452

It must be reminded that equal values of precision, recall and F1 score is neither an error nor paradox, but the true nature of micro-averaging – FP for one class is FN for another, so the total number of FP and FN across the entire dataset will be equal. Similar explanation describes the identical values of micro- and macro-averaged accuracy, as it contains sum of true predictions across all classes. The Fig. S7 presents the ROC curves for micro-averaging.

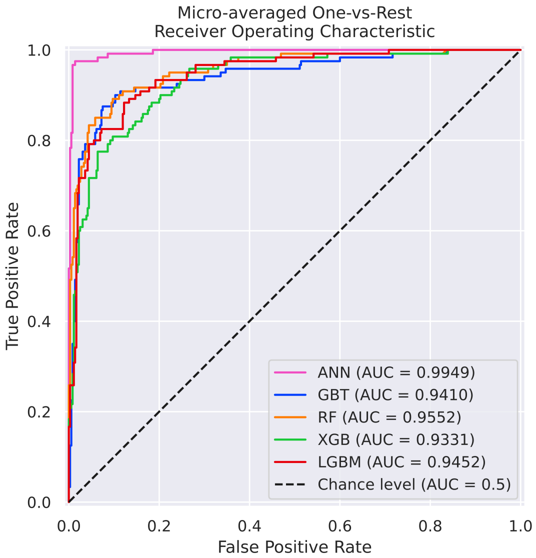

Figure S7. Micro-averaged Receiver Operating Characteristics (ROC) plots for classification predictionson the test dataset, with the corresponding Area Under Curve (AUC) values.

For adsorption energy predictions, three metrics were selected for model evaluation. All three rely on the difference between the predicted and calculated BE.

Mean Absolute Error (MAE) measures the average magnitude of errors in a dataset. MAE is a linear score – all the separate instances are given equal weight in the average.

$$MAE = \frac{\sum_{i=1}^{n} |X_{real} - X_{pred}|}{n}$$

Root Mean Squared Error (RMSE), similarly to MAE, measures the average magnitude of error. The difference is RMSE is a quadratic score, which means that larger deviations are given higher weight in the average.

$$RMSE = \sqrt{\frac{\sum_{i=1}^{n} (X_{real} - X_{pred})^2}{n}}$$

The smaller MAE and RMSE, the closer predicted values are to actual energy values. Difference between the provide insight into error distribution, e.g. RMSE much larger than MAE means that most points are close to the expected value with several cases that strongly deviate from the trend.

The Coefficient of Determination (R^2) provides a measure of how well model replicates the experimental data. With given *y(x)* function, the R^2 can be calculated using two sums of squares formulas: residual sum of squares

$$SS_{res}=\sum_i (y_i-f_i)^2$$

where y_i is measured value and f_i is value according to the fit; and total sum of squares

$$SS_{tot}=\sum_i (y_i-\bar{y})^2$$

where $\bar{y}$ is the arithmetical average of y_i. The R^2 coefficient is then as follows:

$$R^2=1-\frac{SS_{res}}{SS_{tot}}$$

An R^2 value close to 1.0 means that model's predictions are highly consistent and reliable for the physical property being studied.

5. Hyperparameters of the ML

Table S4. Hyperparameters and other characteristics of the machine learning models.

Algorithm	Classification	Regression
Gradient Bosted Trees (GBT)	▪ Loss: Deviance<br>▪ Feature sampling strategy: Square root<br>▪ Number of boosting stages: 274<br>▪ Learning rate (•): 0.224<br>▪ Max trees depth: 9<br>▪ Minimum samples per leaf: 19	▪ Loss: Least Square<br>▪ Feature sampling strategy: Fixed proportion<br>▪ Number of boosting stages: 100<br>▪ Learning rate (•): 0.1<br>▪ Max trees depth: 3<br>▪ Minimum samples per leaf: 1
XGBoost (XGB)	▪ Booster: dart<br>▪ Actual number of trees: 6<br>▪ Max trees depth: 5<br>▪ Learning rate (•): 0.371<br>▪ L1 regularization (α): 0.610<br>▪ L2 regularization (λ): 0.569<br>▪ Min loss reduction to split a leaf (γ): 0.526<br>▪ Min sum of instance weight in a child: 4.262<br>▪ Subsample ratio of the training instance: 0.999<br>▪ Fraction of columns in each tree: 0.526	▪ Booster: gbtree<br>▪ Objective: regression with squared loss<br>▪ Actual number of trees: 123<br>▪ Max trees depth: 3<br>▪ Learning rate (•): 0.2<br>▪ Max delta step: 0<br>▪ L1 regularization (α): 0.0<br>▪ L2 regularization (λ): 1.0<br>▪ Min loss reduction to split a leaf (γ): 0.0<br>▪ Min sum of instance weight in a child: 1.0<br>▪ Subsample ratio of the training instance: 1.0<br>▪ Columns subsample ratio for trees: 1.0<br>▪ Columns subsample ratio for splits / levels: 1.0<br>▪ Balancing of positive and negative weights: 1.0
Random Forest (RF)	▪ Number of trees: 156<br>▪ Max trees depth: 15<br>▪ Min samples per leaf: 2<br>▪ Min samples to split: 6<br>▪ Split quality criterion: Gini<br>▪ Use bootstrap: Yes<br>▪ Feature sampling strategy: prop<br>▪ Used features: 34%	▪ Number of trees: 100<br>▪ Max trees depth: 12<br>▪ Min samples per leaf: 1<br>▪ Min samples to split: 3<br>▪ Split quality criterion: MSE<br>▪ Use bootstrap: Yes<br>▪ Feature sampling strategy: prop<br>▪ Used features: 100%
LightGBM (LGBM)	▪ Booster: gbdt<br>▪ Actual number of trees: 5<br>▪ Maximum number of leaves: 242<br>▪ Learning rate (•): 0.295<br>▪ L1 regularization (α): 0.143<br>▪ L2 regularization (λ): 0.949<br>▪ Minimal gain to perform a split on	▪ Booster: gbdt<br>▪ Actual number of trees: 75<br>▪ Maximum number of leaves: 31<br>▪ Learning rate (•): 0.2<br>▪ L1 regularization (α): 0.0<br>▪ L2 regularization (λ): 0.0<br>▪ Minimal gain to perform a split on a

	a leaf: 0.915<br>▪ Minimum leaf samples: 3<br>▪ Min sum of instance weight in a child: 0.0492<br>▪ Subsample ratio of the training instance: 0.75<br>▪ Columns subsample ratio for trees: 0.611	leaf: 0.0<br>▪ Minimum leaf samples: 10<br>▪ Min sum of instance weight in a child: 0.001<br>▪ Subsample ratio of the training instance: 1<br>▪ Columns subsample ratio for trees: 0.7
Neural Network (ANN)	▪ Number of layers: 5<br>▪ Number of nodes in each layer: 100-100-50-50-4<br>▪ Regularization: dropout (0.2–0.3)<br>▪ Activation function (in hidden layers): leaky ReLU<br>▪ Output activation / Loss function: softmax / categorical cross-entropy<br>▪ Optimizer: Adam<br>▪ Number of epochs for training: up to 2000 (with early stopping)	▪ Number of layers: 5<br>▪ Number of nodes in each layer: 100-100-50-50-1<br>▪ Regularization: dropout (0.2–0.3)<br>▪ Activation function (in hidden layers): swish<br>▪ Output activation / Loss function: linear / MSE<br>▪ Optimizer: RMSProp<br>▪ Number of epochs for training: up to 4000 (with early stopping)

6. Confusion matrices of classification models

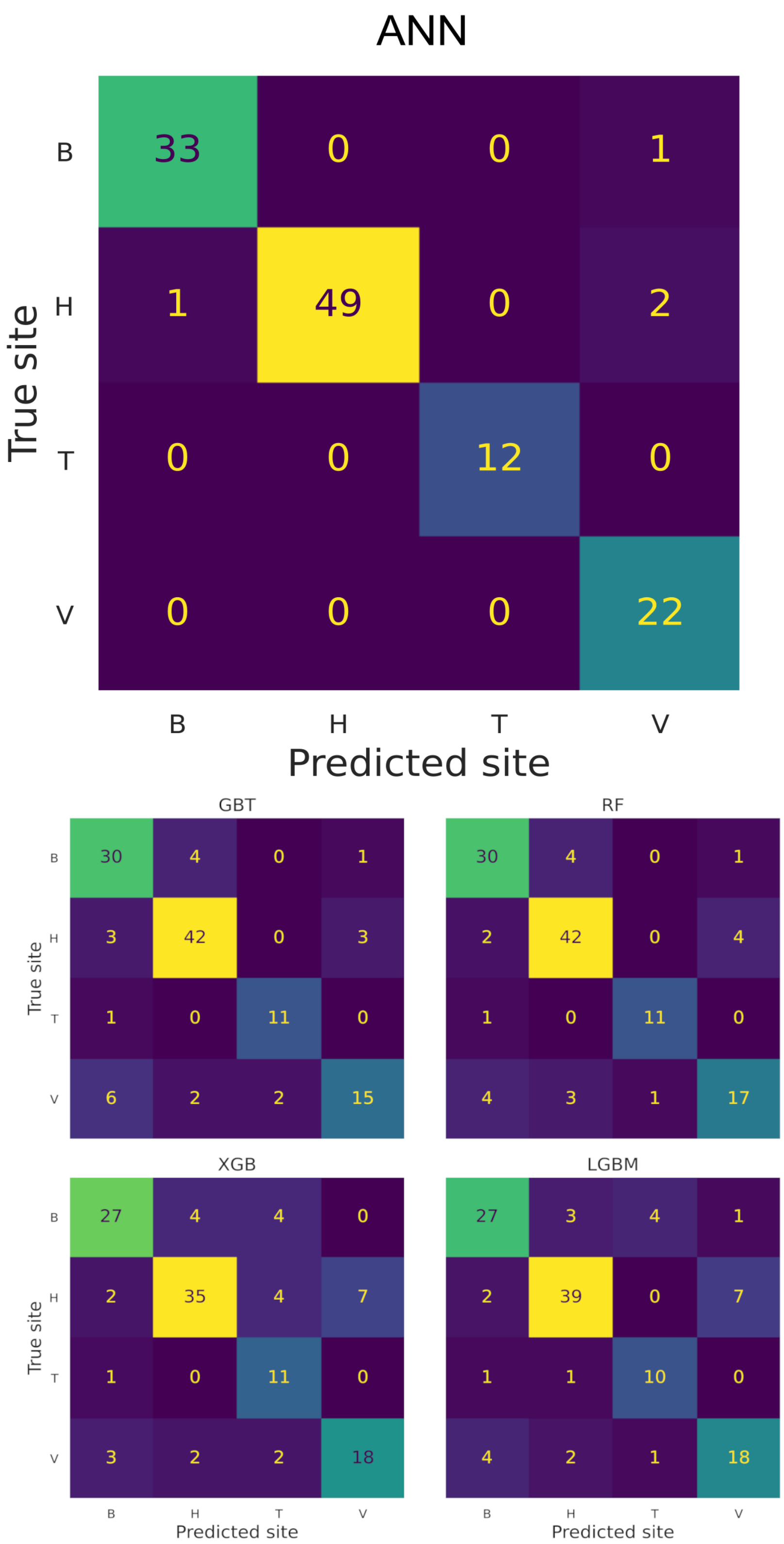

Figure S8. Confusion (error) matrices for classification predictions on the test dataset.

7. Statistical metrics on the validation dataset

Table S5. Classification metrics for ML models, predicting the adsorption site.

	ACC	PPV	TPR	F1	ROC AUC
**ANN**	0.957 (± 0.019)	0.949 (± 0.024)	0.963 (± 0.017)	0.954 (± 0.019)	0.995 (± 0.003)
**GBT**	0.858 (± 0.075)	0.849 (± 0.088)	0.832 (± 0.091)	0.834 (± 0.081)	0.956 (± 0.037)
**RF**	0.856 (± 0.091)	0.834 (± 0.126)	0.831 (± 0.124)	0.828 (± 0.120)	0.941 (± 0.063)
**XGB**	0.783 (± 0.078)	0.747 (± 0.085)	0.786 (± 0.119)	0.756 (± 0.089)	0.932 (± 0.075)
**LGBM**	0.747 (± 0.097)	0.709 (± 0.107)	0.761 (± 0.143)	0.717 (± 0.115)	0.926 (± 0.068)

Table S6. Regression metrics for ML models, predicting the adsorption energy.

	MAE	RMSE	R^2
**ANN**	0.248 (± 0.029)	0.344 (± 0.041)	0.954 (± 0.012)
**XGB**	0.208 (± 0.108)	0.335 (± 0.223)	0.953 (± 0.061)
**RF**	0.241 (± 0.110)	0.385 (± 0.289)	0.937 (± 0.094)
**GBT**	0.269 (± 0.136)	0.417 (± 0.324)	0.926 (± 0.116)
**LGBM**	0.243 (± 0.189)	0.422 (± 0.447)	0.916 (± 0.177)
